# High-stretchability and low-hysteresis strain sensors using origami-inspired 3D mesostructures

**DOI:** 10.1126/sciadv.adh9799

**Published:** 2023-08-25

**Authors:** Xinghao Huang, Liangshu Liu, Yung Hsin Lin, Rui Feng, Yiyang Shen, Yuanning Chang, Hangbo Zhao

**Affiliations:** ^1^ Department of Aerospace and Mechanical Engineering, University of Southern California, Los Angeles, CA 90089, USA.; ^2^ Mork Family Department of Chemical Engineering and Materials Science, University of Southern California, Los Angeles, CA 90089, USA.; ^3^ Ming Hsieh Department of Electrical and Computer Engineering, University of Southern California, Los Angeles, CA 90089, USA.; ^4^ Alfred E. Mann Department of Biomedical Engineering, University of Southern California, Los Angeles, CA 90089, USA.

## Abstract

Stretchable strain sensors are essential for various applications such as wearable electronics, prosthetics, and soft robotics. Strain sensors with high strain range, minimal hysteresis, and fast response speed are highly desirable for accurate measurements of large and dynamic deformations of soft bodies. Current stretchable strain sensors mostly rely on deformable conducting materials, which often have difficulties in achieving these properties simultaneously. In this study, we introduce capacitive strain sensor concepts based on origami-inspired three-dimensional mesoscale electrodes formed by a mechanically guided assembly process. These sensors exhibit up to 200% stretchability with 1.2% degree of hysteresis, <22 ms response time, small sensing area (~5 mm^2^), and directional strain responses. To showcase potential applications, we demonstrate the use of distributed strain sensors for measuring multimodal deformations of a soft continuum arm.

## Teaser

3D foldable electrode-based capacitive sensors can provide high stretchability and low hysteresis for strain sensing.

## INTRODUCTION

Accurate measurement of large deformations is a critical requirement in a broad range of engineering applications, such as body motion tracking for healthcare (*1*, *2*), proprioception and control of soft robotics (*3*), and human-machine interfaces (*4*). Desirable sensor performance includes large strain range, low hysteresis, high sensitivity and linearity, high stability and robustness, minimal size and weight, and user-friendly data acquisition and processing. Among the various strain sensing mechanisms (*5*), resistive and capacitive sensing have been studied extensively because of their ease of fabrication and signal readout. Commonly used strategies for these stretchable resistive and capacitive strain sensors include using intrinsically conductive polymers or combining stretchable matrices with active conductive components. Examples include conducting polymers (*6*), ionic liquids (*7*, *8*), liquid metals (*9*), nanowires (*10*), and nanocomposites with conductive nanoparticles dispersed in polymer matrices (*11*). Despite their relatively large mechanical stretchability, many of these sensors exhibit unfavorable characteristics, such as electromechanical hysteresis, slow recovery, and lack of long-term stability. For example, conductive polymer nanocomposites often suffer from relatively large hysteresis in resistive strain sensing due to irreversible changes in the morphology of the conductive fillers during deformation (*12*, *13*); liquid metal–based sensors have challenges in achieving fast response, sensor miniaturization, and long-term stability due to the interfacial properties of liquid metals (*14*).

As an alternative approach, structure designs capable of mechanical stretching can provide means of achieving stretchable strain sensing without relying on stretchable conductive materials. These designs include wavy structures, fractal designs, twisted or helical structures, open-mesh structures, and origami- and kirigami-inspired structures (*15*–*23*). For example, parallel-plate capacitive strain sensors using wrinkled ultrathin gold film electrodes exhibit high sensitivity and linearity with small hysteresis, but with limited stretchability (140%) (*18*). Similar wrinkled metal thin-film electrodes on microstructured elastomeric surfaces provide increased stretchability (up to 250% strain) but suffer from uncontrollable crack propagation in the metal thin films at large strain (*19*). Auxetic structures with engineered cuts can provide stretchability for parallel-plate capacitive sensors but with limited stretchability (50%) (*20*). Miura-patterned, foil-based sensors are capable of measuring capacitance changes during folding and unfolding with a low strain range (20%) (*21*). Stretchable strain sensors with large strain range, low hysteresis, and fast response are highly desirable, especially for accurate measurement of large and dynamic deformations.

Here, we introduce a three-dimensional (3D) strain sensor design using a deformable non-parallel plate configuration. The 3D electrode structures take the form of origami-inspired, folded plates fabricated by a mechanically guided assembly process. The assembly process geometrically transforms 2D electrode patterns into out-of-plane, folded plates with adjustable folding angles, which are covalently bonded to a silicone elastomeric substrate. A silicone cover encloses the 3D electrodes within a compartment filled with liquid glycerol. The resulting system can convert large substrate deformations into changing angles between the folded 3D plate electrodes, thereby providing large stretchability (200%) with ultralow hysteresis (1.2%), high repeatability (over 700 stretching cycles with 100% strain), fast response time (<22 ms), and small sensor footprint (sensing area ~ 5 mm^2^). Furthermore, the unique 3D electrode design also enables compressive strain sensing and directional strain responses, which are distinct from existing stretchable strain sensors. The millimeter-scale sensing area on a thin substrate allows for the attachment of the sensor on soft bodies using a simple stick-on method, separating the sensor fabrication and implementation on target objects. These features are particularly attractive for distributed sensing of soft body deformation, as demonstrated with a soft continuum arm. This concept of origami-inspired 3D stretchable capacitive sensors is potentially useful in wearable sensors, prosthetics, soft robotics, and human-machine interfaces.

## RESULTS

### Working mechanism and fabrication of the strain sensor

**Fig. 1.**
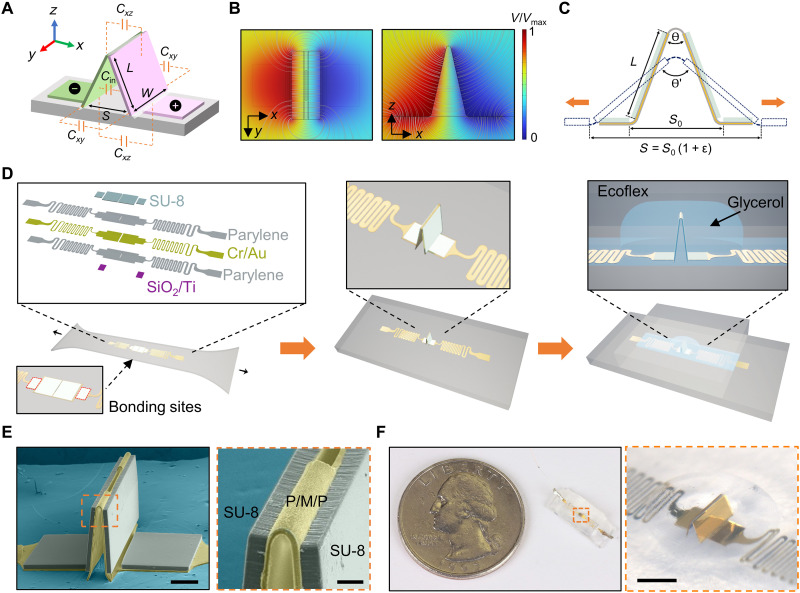
Design, sensing mechanism, and construction of origami-inspired capacitive strain sensors. (**A**) Schematic illustration of the non-parallel-plate configuration and sensing mechanism. (**B**) Top-down and side views of the simulated electric field for the 3D non-parallel plates in the strain sensor. (**C**) Schematic illustration of the unfolding of the 3D electrodes under stretching. (**D**) Schematic illustration of the sensor fabrication process including bonding the 2D precursor to a prestretched elastomeric substrate, compressive buckling of the electrodes, and encapsulation of the 3D electrodes inside a glycerol-filled compartment. Bonding only occurs at the bonding sites encircled by the red dashed lines. (**E**) False-color SEM images showing origami-inspired 3D mesoscale electrodes. P/M/P represents the parylene/metal/parylene trilayer. (**F**) Optical image of a strain sensor and a close-up view of the sensing element. Scale bars, 200 μm in (E) (50 μm in inset) and 1 mm in (F).

The capacitive strain sensor design is based on a pair of foldable 3D mesoscale electrodes inspired by origami—the art of paper folding (*24*). The basic structure features a multi-panel thin film in a triangular shape on a stretchable dielectric substrate (Fig. 1A). Each electrode (colored in pink and green) consists of two flat panels: one bonded to the substrate and the other free to rotate. Thin, crease-like structures connect the two panels of each electrode and the top edges of the rotatable panels. Biasing the two electrodes generates a 3D electric field with field lines within and outside the triangular-shaped plates, as well as those crossing the substrate from the flat portions of the two electrodes. Figure 1B shows the side and top-down views of the 3D electric field from finite element analysis (FEA) with the electric field lines highlighted. Details of the electromagnetic FEA appear in Materials and Methods. The capacitance measured between the two electrodes includes the following main components: inner capacitance between the two angled panels within the triangle *C*_in_ and capacitances between the two electrodes outside the triangle in the *xy* plane *C_xy_* and in the *xz* plane *C_xz_*. The total capacitance between the two electrodes, *C*_total_, is approximately the sum of these components due to their parallel configuration:

$$C_{\mathrm{total}}\approx C_{\mathrm{in}}+C_{xy}+C_{xz}$$

*C*_in_, *C_xy_*, and *C_xz_* can be analytically approximated using a capacitance model of non-parallel plates via a conformal mapping approach (*25*, *26*). The Supplementary Materials provide detailed analysis.

Partial bonding of the electrodes to the substrate and foldable creases allow the 3D electrodes to be stretched or compressed along the *x* direction, which changes the distance between the two panels bonded to the substrate and the folding angles of the three creases. Fig. 1C schematically shows the stretching of the 3D electrodes under strain ɛ and the increase of the top crease angle from θ to θ′ due to unfolding of the panels. Analytical approximations indicate decreases in *C*_in_, *C_xy_*, and *C_xz_* with increasing top crease angle, causing a decrease in the total capacitance *C*_total_ (see the Supplementary Materials). As a result, stretching or compressing the substrate of the 3D electrodes changes the 3D electric field between the biased electrodes, leading to an increase or decrease in the total measured capacitance *C*_total_.

On the basis of the strain sensing mechanism described above, we realize the 3D electrode design concept by leveraging recent advances in the mechanically guided assembly of 3D mesostructures (*27*–*30*). Fig. 1D schematically shows the key steps in the fabrication process. The process begins with the fabrication of a planar multilayered structure, referred to as the 2D precursor. The 2D precursor includes a bottom parylene C layer (5 μm in thickness), a lithographically patterned metal layer (chromium/gold, 25/200 nm in thickness), and another parylene C layer (5 μm in thickness) above it. Patterning and etching the parylene layers create a parylene/metal/parylene pattern that defines the multipanel electrodes and a pair of narrow (~100 μm) serpentine traces. These traces serve as highly stretchable electrical interconnects between the electrodes and external electronics. A thick (35 to 40 μm in thickness), photodefinable epoxy (SU-8 25) coating the top parylene C layer provides stiffening at four patterned, rectangular areas. Coverage of this SU-8 layer increases the bending stiffness by a factor of approximately 130 to 175 compared to uncovered regions, thereby forming panel-crease structures. A slit design in the parylene/metal/parylene crease connecting the two electrodes further reduces the bending stiffness of the crease. Deposition of a bilayer of Ti/SiO_2_ (15 nm/50 nm in thickness) on the backside of the 2D precursor through a shadow mask forms sites for covalent bonding to a uniaxially prestretched elastomer substrate (Ecoflex 00-31). Releasing the prestrain imparts compressive forces that induce buckling of the unbonded regions of the 2D precursor and subsequent folding deformations in the creases. Details of the 2D precursor fabrication and 3D assembly processes appear in Materials and Methods. Figure S1 shows the layouts of the 2D precursor. As a result of the mechanically guided assembly process, the two panels form a triangular shape (Fig. 1D, middle), with the gap between the two bonding sites *S*_0_ determined by the electrode length *L* and the prestrain ɛ_pre_: $S_{0}=\frac{2L}{1+\varepsilon_{\mathrm{pre}}}$. Fig. 1E presents the scanning electron microscopy (SEM) images of a 3D mesoscale electrode structure after mechanically guided assembly. Because of the small bonding site area (~500 μm × 650 μm) and the high bending stiffness of the SU-8 layer, the electrodes bonded on the substrate remain flat without buckling.

Following the formation of the 3D foldable electrodes, bonding a top silicone cover to the silicone substrate encapsulates the 3D electrodes. The cover layer, formed by replica molding (fig. S2), includes a dome-shaped compartment and two channels (2.5 mm in width and 0.3 mm in thickness) to accommodate deformations of the 3D electrodes and the serpentine interconnects, respectively. Filling the compartment and channels with nonvolatile liquid glycerol increases the permittivity of the dielectric medium [ɛ = 40 to 50 at 25°C (*31*)] between the electrodes while maintaining sensor flexibility. The liquid glycerol dielectric increases the baseline capacitance of the sensor (*32*), leading to an increased signal-to-noise ratio as compared to the air dielectric (fig. S3). Electrical connection to the serpentine interconnects and sealing of the fluid channel complete the device fabrication. Fig. 1F shows a completed sensor device with an electrode length of 500 μm, width of 1000 μm, and 3D structure height of approximately 560 μm. The active sensing area—the 3D electrode area—is approximately 5 mm^2^. The serpentine interconnects have a width of 110 μm and an adjustable length. The overall dimension of the sensor is customizable depending on specific applications. For convenience in mechanical testing, a typical sensor adopts a strip shape with a width of 10 mm and a length of 10 to 50 mm (Fig. 1F and fig. S4).

### Sensor design parameters

**Fig. 2.**
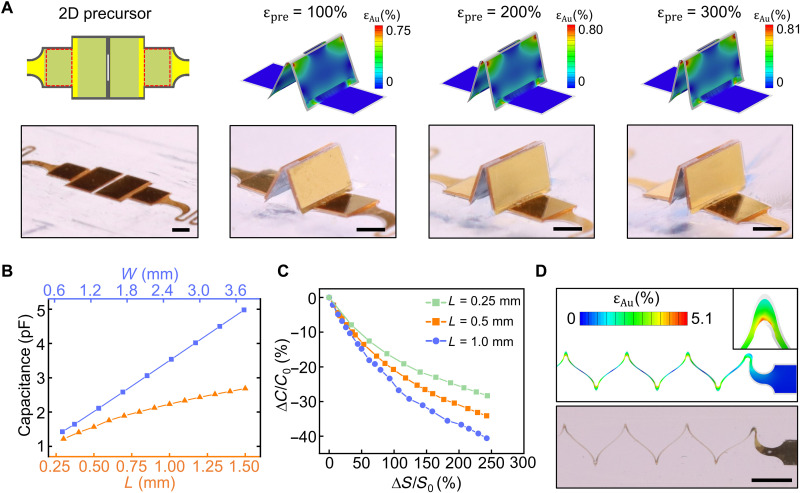
Basic sensor structure designs and the mechanics of sensor formation and stretching. (**A**) FEA and experimental images of origami-inspired 3D non-parallel plates with different folding angles, formed by mechanically guided assembly. The regions encircled by red dashed lines represent bonding sites. The contours in the FEA images display the strain in the gold layer. (**B**) Influences of the electrode length *L* and width *W* on the baseline capacitance of the sensor (300% prestrain). (**C**) Relative capacitance change of a sensor with different electrode lengths and applied strain between the bonding sites (300% prestrain, *W* = 1 mm). (**D**) FEA and experimental images of serpentine electrical interconnects under stretching, with the strain in the gold layer shown in the FEA contours. Scale bars, 500 μm in (A) and 1 mm in (D).

Key sensor design parameters include the prestrain applied during 3D assembly, electrode dimensions, and the serpentine layout. The prestrain ɛ_pre_ dictates the folding angle θ of the 3D electrodes and the corresponding distance between the bonding sites. FEA that captures the mechanical properties of the constituent materials of the 2D precursor can provide accurate predictions of the geometric transformation from 2D to 3D during the assembly process and the resulting structure of the 3D electrodes. The shapes of the resulting 3D electrodes of a representative design with different prestrain levels predicated by FEA agree well with experimental results (Fig. 2A). Similar results for an electrode design with a larger electrode length (1 mm) appear in fig. S5. Quantitative measurements of the heights of the triangular-shaped electrodes from FEA and experiments (fig. S6) indicate high agreement with less than 6.5% relative error (table S1). The errors are likely due to imperfections of the fabrication process, such as slight misalignment of the bonding sites with the electrode pattern. Excessive bonding can occur after the assembly process but quickly disappears because of partial delamination, resulting in a stable 3D structure after 1 to 2 days (fig. S7). In addition to geometric modeling, FEA also provides quantitative strain distributions in the constituent materials, such as the distribution of the strain in the Au electrode layer (Fig. 2A). Most of the Au thin film exhibits strain levels well below the yield strain of deposited Au thin films (*33*) even at ɛ_pre_= 300%, owing to the position of the Au layer near the mechanical neural plane of the parylene/metal/parylene trilayer at the creases and the panel designs. The maximum equivalent strain appears near the corners of the top crease, which may be slightly above the yield strain but highly localized. The prestrain also sets an upper bound for the maximum uniaxial strain the sensor can measure (ɛ_max_* = *ɛ_pre_), beyond which the electrodes are fully unfolded and susceptible to fracture upon further stretching.

The electrode dimensions influence the initial capacitance of the 3D electrodes and the capacitance change during the folding/unfolding processes. With fixed prestrain, the capacitance increases with both increased electrode width *W* and length *L*. FEA indicates a linear trend for electrode width, but the slope is smaller for increased electrode length above a certain value with the crease length fixed (approximately 1 mm for 80-μm crease length under 300% prestrain; Fig. 2B). Fig. 2C shows the simulated relative capacitance change of sensors with different electrode lengths as a function of applied strain at the bonding sites, ∆*S*/*S*_0_. Sensors with an electrode length of 1.0 mm have a larger capacitance change compared to those with shorter electrode lengths, such as 0.5 and 0.25 mm. The gauge factor of the strain sensor, defined as $\mathrm{GF}=\frac{\Delta C/C_{0}}{\varepsilon }$, is −0.11, −0.21, and −0.25 for *L* = 0.1, 0.5, and 1.0 mm, respectively, at 100% local strain. While larger electrode lengths result in larger baseline capacitance and higher strain sensitivity, submillimeter electrode lengths are typically chosen for sensor miniaturization.

Serpentine metal traces provide stretchable electrical interconnects, which is critical for robust electrical sensor readout. We adopt a serpentine pattern with 110-μm linewidth, 900-μm length, and 180° arc angle. The serpentine traces can accommodate large uniaxial stretching without fracture due to their out-of-plane buckling and twisting inside the channels. The placement of the Au layer in the mechanical neural plane of the parylene/metal/parylene trilayer also minimizes strain caused by bending. Fig. 2D shows the comparison between FEA and experimental results of the serpentine traces upon 200% stretching, indicating a strong agreement between the stretched shapes. The maximum equivalent strain in the Au layer is 5.1% when 200% stretching is applied, located only at the inner corners of the serpentine. Most of the Au serpentine interconnects remain within the fracture limit (*34*, *35*), consistent with experimental observations that 200% stretching does not cause electrical failure of the serpentine interconnects.

### Sensor characterization

**Fig. 3.**
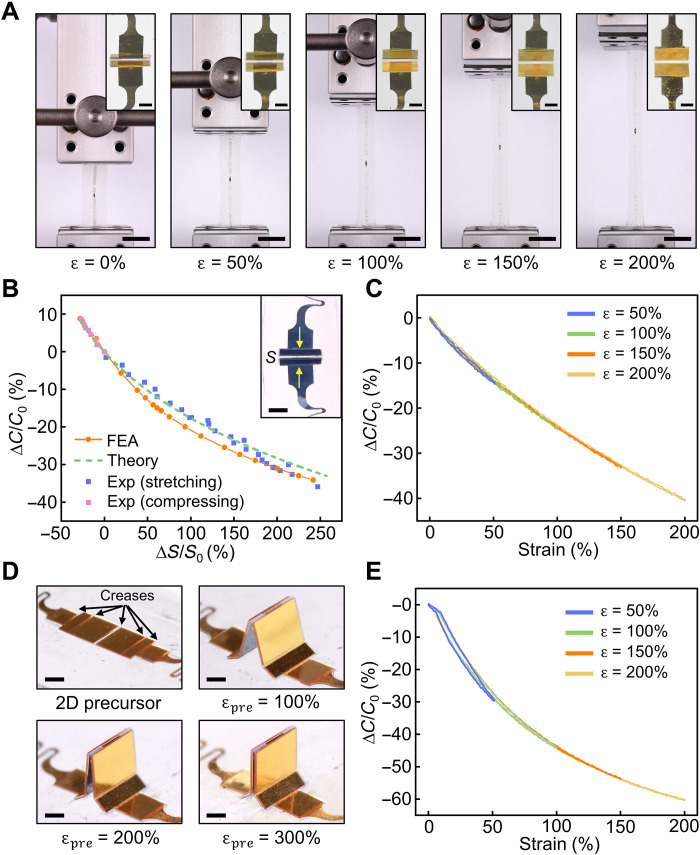
Characterization of the strain sensing range and the corresponding DH of the sensors. (**A**) Optical images of a sensor under uniaxial stretching at 0, 50, 100, 150, and 200%. Insets show close-up images of the respective 3D electrodes. (**B**) Simulation, analytical, and experimental results for the relative change in bonding site distance and the corresponding relative capacitance change of a basic, three-crease electrode design (*L* = 0.5 mm). *C*_0_ denotes the capacitance with a bonding site distance *S* = 210 μm. (**C**) Relative capacitance change of a representative sensor (*L* = 0.5 mm) with a basic, three-crease electrode design during uniaxial loading and unloading at 50, 100, 150, and 200% applied strain. (**D**) Optical images of the 2D precursor of a five-crease electrode design and the resulting 3D electrodes formed with different prestrain. (**E**) Relative capacitance change of the five-crease strain sensor (300% prestrain) during uniaxial loading and unloading at 50, 100, 150, and 200% applied strain. Scale bars, 1 cm in (A) (300 μm in insets) and 500 μm in (B) and (D).

Following the abovementioned design considerations, 3D capacitive strain sensors can measure large strain. Fig. 3A shows images of a sensor in a long (40 mm in length) strip subjected to uniaxial stretching with nominal strains of 0, 50, 100, 150, and 200% applied to the strip. Microscopic images (insets) capture the unfolding of the 3D angled electrodes at the center of the sensor strip. Fig. 3B presents measurements of the relative capacitance change ∆*C*/*C*_0_ as a function of the relative change in the bonding site distance, i.e., the local strain at the bonding sites ∆*S*/*S*_0_. *C*_0_ represents the sensor capacitance without deformation, and experimental measurements involve both stretching and compressing the sensor. Results from FEA of the 3D electric field agree well with experimental measurements, with discrepancies likely originating from imperfections of the 3D electrode mesostructures. Results from analytical modeling agree well with the experiments and simulation results. Stretching tests for sensors with electrode length of 1 mm (fig. S8) also show similar decreasing trends among simulation, measurement, and analytical results. The relative capacitance change from analytical results is smaller at large strain as compared to simulation and experiment results. This is likely caused by the reduced gap between the Ecoflex encapsulation layer and the 3D electrodes for this larger electrode design (details appear in the Supplementary Materials).

Degree of hysteresis (DH) is an important performance parameter for strain sensors as it affects their accuracy and repeatability. Since strain sensing of our sensors relies almost entirely on the geometric changes of the 3D electrode configuration on an elastomeric substrate, the sensors exhibit low levels of DH. Fig. 3C shows the loading and unloading curves of a representative sensor (*L* = 0.5 mm, *W* = 1.0 mm) stretched up to 200% strain at a strain rate of 10% s^−1^. Movie S1 shows synchronized footage between capacitance change and sensor stretching. The sensor shows GF = −0.25 at 100% strain and GF = −0.20 at 200% strain. The sensor response is highly consistent at different strains. The DH values measured from the loading/unloading loops are 4.88 ± 1.67% at ɛ = 50%, 2.35 ± 0.63% at ɛ = 100%, 1.84 ± 0.68% at ɛ = 150%, and 1.19% ± 0.58% at ɛ = 200% (each measured from five sensors). Given the reversible nature of folding/unfolding deformations of the 3D electrodes, the measured sensor hysteresis mainly originates from the viscoelasticity of the silicone elastomeric substrate (*36*). The decreasing DH with increasing strain range suggests that prestretching the sensor before strain sensing leads to reduced DH for the same applied strain. For example, the measured DH from a sensor without prestretching for 100% applied strain is 3.32%, and it decreases to 1.50% for 10% prestretching and 0.79% for 20% prestretching (fig. S9).

The choice of the representative electrode size balances sensor miniaturization and performance. Figure S10 shows a sensor design with *L* = 0.25 mm and *W* = 0.55 mm. This reduction in electrode dimensions leads to smaller baseline sensor capacitance, making it more susceptible to noise and parasitic capacitance. Further miniaturization also presents challenges in precise control of the bonding regions between the electrodes and the substrate, often leading to increased hysteresis.

The design versatility of the 2D precursors enables the creation of more complex, origami- or kirigami-inspired 3D electrodes. Fig. 3D highlights an example of a multi-crease structure where two additional creases are added to the large panels. The corresponding 2D precursor design appears in fig. S11. Different prestrain before the compressive buckling process leads to distinct 3D shapes, from multisegment triangular shapes to fully closed, vertically aligned plates due to the presence of the additional foldable panels next to the middle panels. This contrasts with the basic three-crease design in Fig. 2A, where 300% prestrain results in an angle of 22° to 24° between the two folded plates. Fig. 3E shows the relative capacitance change of a representative sensor with the five-crease electrode design and 300% prestrain. Stretching of a fully closed electrode configuration causes a faster decrease in *C*_in_, the main component of the total capacitance, due to a smaller folding angle θ. As a result, this multi-crease electrode design leads to higher sensitivity (GF = −0.44 at 100% strain and GF = −0.30 at 200% strain) as compared to that of the basic design.

Besides different crease designs, variations in the electrode shape offer additional sensor design options. Figure S12 show two three-crease structures with trapezoidal-shaped foldable electrodes (electrode length *L* = 0.5 mm and *L* = 1.0 mm). The sensor with *L* = 0.5 mm exhibits similar capacitance responses (GF = −0.26 at 100% strain) as that based on the rectangular-shaped electrode. In contrast, the sensor with *L* = 1.0 mm shows higher sensitivity (GF = −0.39 at 100% strain) than that (GF = −0.36 at 100% strain) of the sensor with rectangular electrode design and the same electrode length, likely due to the relatively larger contribution of the upper portion of the folded electrodes in the total capacitance change. Table S2 summarizes performance parameters of sensors with different electrode designs under 100% strain. Increase in *L* from 0.25 to 1 mm leads to increased sensitivity, with slight decrease in the linearity due to more fringe capacitance change introduced at larger bonding site gaps. Larger electrode area increases the baseline capacitance, which reduces the influence of parasitic capacitance and measurement noise.

**Fig. 4.**
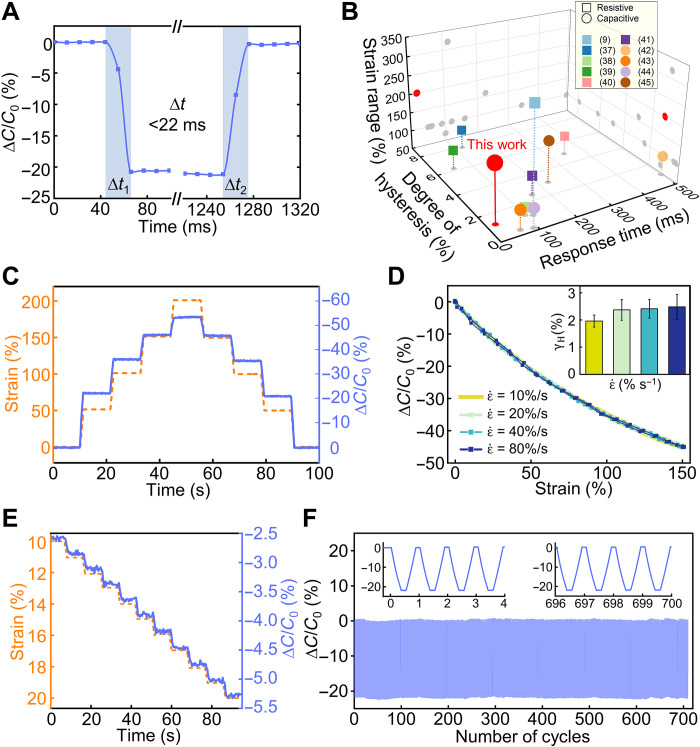
Characterization of the sensor performance including response and recovery time, strain rate, strain resolution, and sensor repeatability. (**A**) Real-time response of a strain sensor subjected to a quick 100% step strain, showing the response time and recovery time. (**B**) Comparison of the sensor in this work with previously reported strain sensors in the sensor strain range, DH, and response time. (**C**) Capacitance response of a representative sensor (*L *= 1 mm) under a series of step-up strain of 50% to a maximum of 200% followed by step-down strain to the initial state. (**D**) Capacitance response of a sensor (*L *= 1 mm) stretched to 150% strain at strain rates of 10, 20, 40, and 80% s^−1^. The bar plot shows the hysteresis at different strain rates. (**E**) Relative capacitance change of a representative sensor (*L *= 0.5 mm) under increasing strain with 1% strain increments. (**F**) Relative capacitance change of a representative sensor (*L *= 0.5 mm) over 700 loading/unloading cycles at 100% strain, with close-up views at the beginning and end of the test.

In addition to the large strain range and small hysteresis, these foldable electrode-based capacitive sensors have other attractive features for strain sensing. Fig. 4 presents several features measured from sensors with the basic triangular electrode design. Without the presence of viscoelasticity in the active sensing materials, the sensor has fast response to deformation. A quick stretching action (100% strain) applied to the sensor using a custom actuation setup (fig. S13A) synchronized with capacitance measurement allows for the characterization of the sensor response time, defined here as the time interval between the sensor output signal change and the moment a stable sensor output value is reached under a step input. The sensor shows both response time ∆*t*_1_ and the recovery time ∆*t*_2_ of less than 22 ms, estimated from the sampling rate of capacitance measurement (Fig. 4A). Capacitance responses to multiple quick stretching and releasing actions demonstrate the consistency in the sensor’s fast response and recovery time (fig. S13B).

The combination of large strain range (200%) with low DH (1.19%) and fast response (<22 ms) distinguishes our strain sensor from other stretchable strain sensors. Fig. 4B and table S3 compare our sensors with other selected resistive and capacitive strain sensors in the literature on the three important sensor performance parameters (*9*, *37*–*45*). In this comparison, we only include examples with all these three parameters reported. Our sensors have one of the lowest levels of DH and response time among all strain sensors with large (>100%) strain range including examples not shown in Fig. 4B (*7*, *18*, *46*–*48*), which are critical for accurate and dynamic strain sensing. The strain range of our sensors is also large among capacitive strain sensors owing to the unique 3D mesostructured electrode design as compared to conventional parallel-plate capacitors. While there are resistive and capacitive strain sensors that have larger strain range (*9*, *48*, *49*), they typically suffer from relatively large DH or response time due to their sensing mechanism and materials used. The strain range of our sensors can be extended readily to be above 200% by increasing the prestrain in the mechanically guided assembly process for forming the 3D electrodes and optimizing the serpentine interconnects.

The low hysteresis and small response/recovery time enable the sensors to measure fast, dynamically changing deformations. Fig. 4C shows an exemplary sensor response to a step-and-hold test where the sensor is subject to alternating stepwise stretching with a 50% strain step size at 10 mm/s (42% s^−1^) and holding (10 s). The applied strain increases to a maximum of 200% strain and then decreases to zero. No obvious creeping effect is observed in the sensor readout throughout the test. Similar tests for other stretching speeds appear in fig. S14. More detailed sensor responses to different strain rates appear in Fig. 4D. The largely overlapping response curves indicate velocity-insensitive sensor performance. Because of higher energy dissipation at high strain rates (*50*, *51*), hysteresis increases from 1.96 to 2.48% when the strain rate increases from 10 to 80% s^−1^. The maximum difference at any specific strain is within 1.0% in ∆*C*/*C*_0 _when the sensor is stretched at 150% strain with strain rates of 10, 20, 40, and 80% s^−1^.

The sensor’s low hysteresis and stable triangular electrode structure allow the detection of small strain changes (<1%) over 100% strain range using standard capacitance measurement units (Fig. 4E and fig. S15). For each 1% strain increment, the average relative capacitance change is 0.22% and the signal-to-noise ratio is approximately 60 (noise signal appears in fig. S16), indicating distinguishable capacitance signals for small strain changes. The sensor exhibits repeatable responses to 100% strain over 700 loading and unloading cycles (Fig. 4F). The maximum variation in the relative capacitance change during these cycles is 0.8%, owing to the structure stability of the 3D electrodes and the robust bonding of the electrodes to the silicone substrate. The sensor also exhibits stable performance over 15 days. The hysteresis remains mostly unchanged with a slight decrease (~5%) in sensitivity (fig. S17).

Normal and shear stress applied to the active sensing area may deform the angled electrodes, resulting in capacitance changes that can confound strain sensing. Insensitivity to these forces may be important for strain sensors used in environments that involve contacts with the sensors, such as in wearable devices. The presence of the top silicone cover and the liquid glycerol helps mitigate this effect by reducing the deformation of the 3D electrodes under normal or shear stress. Characterization and discussion of the influence of normal pressure on strain sensing appear in note S2 and fig. S18. Detailed characterization of the sensor’s mechanical robustness against collisions and abrasions is available in note S3, fig. S19, and movie S2.

Electromagnetic interference could also affect the capacitance measurement due to the presence of parasitic capacitance. Insensitivity to electromagnetic interference is crucial for applications where the sensors are in close proximity to electromagnetic signals or electrostatic discharge. Our sensor without electromagnetic shielding shows noise levels of approximately 8% relative capacitance change when a human finger is hovering above or pressing the sensor (fig. S20). The use of shielding layers enclosing the sensor can reduce the level of interference. Bonding two layers of low-cost, conductive fabrics with laser-cut patterns on the top and bottom surfaces of the capacitive strain sensor completes the shielding process. The kirigami-inspired cuts increase the stretchability of the shielding layers. The addition of the shielding layers reduces the magnitude of the interference to 1 to 2% relative capacitance change, which is much smaller than the strain-induced capacitance changes for large strain sensing. The shielding layers also improve the signal quality by reducing the noise level from 0.182 to 0.05% (fig. S20D). Further reduction is possible by using stretchable conductors such as conductive nanocomposites or liquid metal–filled silicone as shielding materials to provide better coverage of the sensor while maintaining large stretchability (*52*).

### Proof-of-concept demonstration in sensing soft arm deformations

Stretchable strain sensors can measure relatively large deformations and have been used in soft robots to sense their shapes and deformations. Distributed strain sensors can be integrated seamlessly with soft bodies to compute strain distributions for 3D reconstruction (*53*, *54*), providing a solution to the proprioception of soft robots in arbitrary environmental settings. However, strain sensors with high hysteresis or low stretchability could limit proprioception capability (*13*, *55*). Another challenge of soft robot proprioception is the decomposition of the multimodal deformations of soft robots, such as stretching/compression, bending, twisting, and shear, which could exist simultaneously. Strain sensors capable of multidirectional sensing are highly desirable for applications where deformations could occur in all directions (*23*, *56*).

**Fig. 5.**
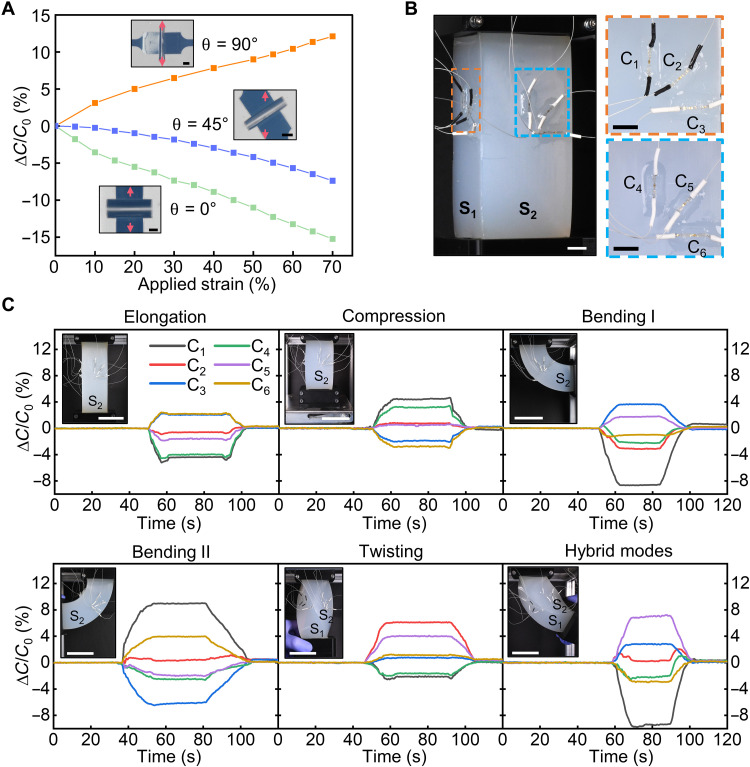
Sensing deformations of a soft continuum arm using the sensor. (**A**) Relative capacitance change of a sensor attached to a silicone slab at different angles (0°, 45°, and 90°) with respect to a uniaxial stretching of 70% strain. Insets show top-down optical images of the 3D electrodes at 70% strain. (**B**) Configuration of two groups of sensors in rosette patterns attached to the two surfaces of a soft continuum arm. (**C**) Distributed sensor responses during elongation, compression, bending, twisting, and hybrid deformation modes of the soft arm. The *S*_1_ and *S*_2_ faces in the insets correspond to those shown in (B). Scale bars, 250 μm in (A), 1 cm in (B) (1 cm in insets), and 5 cm in (C).

The 3D angled electrodes in our capacitive sensors can unfold or fold in response to stretching or compression along the direction of the bonding sites (typically aligns with the longitudinal direction of the sensor strip), respectively, causing decrease or increase in the capacitance (Fig. 3B). Because of the asymmetric structure of the 3D electrodes, the sensor exhibits direction-dependent capacitance changes under stretching applied at different angles with respect to the sensor strip’s longitudinal direction. When a sensor strip is attached to a slab of silicone acting as the target surface with the sensor strip aligned with the stretching direction (θ = 0°), the 3D electrodes unfold (fig. S21), thereby increasing the bonding site distance and decreasing the capacitance. When the sensor strip is perpendicular to the stretching direction (θ = 90°), stretching of the target silicone causes compression of the sensor strip due to the Poisson’s effect of the elastomeric substrate. This compression decreases the bonding site distance, thereby increasing the capacitance. The sensor can measure compressive strain from 0 to −45% despite out-of-plane deformations of certain regions in the serpentine interconnects (fig. S22). When the stretching direction is at an intermediate angle such as θ = 45° with respect to the sensor strip, stretching of the target silicone leads to slight shear movement between bonding sites, which can cause twisting of the 3D electrodes. The resulting capacitance decrease (Fig. 5A) is less than the sensor response at θ = 0° and can also be approximated by FEA simulation (fig. S23).

These directional sensor responses, combined with a rosette configuration, can support measurements of localized strain in different directions. As an example, Fig. 5B shows two three-element (0°/45°/90°) rectangular rosettes attached to two adjacent surfaces (*S*_1_ and *S*_2_) of a soft continuum arm made of silicone using a stick-on method (details appear in Materials and Methods). The rosette configuration can measure in-plane strain components along two perpendicular axes and the shear strain. *C*_1_ to *C*_6_ represent the capacitance values of the six 3D capacitive strain sensors attached to the arm. Deformations of the soft continuum arm in different modes generate different local strain at the sensor locations, inducing deformation-dependent sensor responses (Fig. 5C). Elongation of the arm along its longitudinal direction leads to relatively large capacitance decrease in *C*_1_ and *C*_4_, small capacitance decreases in *C*_2_ and *C*_5_, and large capacitance increases in *C*_3_ and *C*_6_, consistent with the results in Fig. 5A. Compression along the arm longitudinal direction generates opposite capacitance changes. The capacitance changes of the distributed sensors allow for the calculation of the local strain using the relationship between capacitance and bonding site distance shown in Fig. 3B. The calculated local strain values from the sensor responses are compared with those from FEA that captures the geometry and mechanics of the silicone arm and the sensors, as well as the locations and orientation of the sensors (fig. S24). The calculated local strain values from the sensors agree reasonably well with those from the FEA. The two rosettes on *S*_1_ and *S*_2_ exhibit different responses in the three groups, *C*_1_ and *C*_4_, *C*_2_ and *C*_5_, and *C*_3_ and *C*_6_, mainly because of the manual loading processes that create nonuniform strain distributions for elongation and compression. Differences in the sensor attachment and sensor location estimations from imaging can also contribute to the observed differences of sensor responses.

More complex, asymmetrical deformation modes can lead to distinct sensor responses from face *S*_1_ and *S*_2_. Bending the arm generates significant tensile strain on the outer surface and compressive strain on the inner surface, captured by the strain sensor rosettes. Twisting the arm counterclockwise (Fig. 5C) and clockwise (fig. S25) yields the largest capacitance changes in sensors oriented at θ = 45° as twisting induced substantial shear strain in the 45° direction on the surface. Simultaneous multiple deformation modes give rise to intricate sensor responses depending on the directions and magnitudes of the deformations.

This simple demonstration of our strain sensors on a soft continuum arm highlights the potentials for the sensors to accurately measure local strain for large deformations, owing to the large strain range and low hysteresis. The high repeatability, capability of small strain detection, and rapid sensor response are all highly desirable for sensing soft robot deformation. By strategically distributing these miniaturized sensors on target surfaces, unique sensor response patterns can be generated to characterize complex deformation states. An example of classifying nine different single-mode deformations using commonly used machine learning models appears in note S4 and fig. S26. The measured data from the distributed sensor network can also be used for accurate reconstruction of soft robots’ 3D shapes through physics-guided (*57*) or data-driven modeling (*13*, *55*) approaches.

## DISCUSSION

In summary, we have presented design concepts and methods for integrating 3D foldable, mesoscale electrodes with elastomeric substrates for capacitive strain sensing. The stretchability of the substrates and the reversible folding/unfolding of the electrodes allow for a large strain sensing range with minimal hysteresis. Comprehensive sensor characterizations are performed, including the sensor stretchability, hysteresis, response and recovery time, strain resolution, repeatability, and directional strain sensing. Analytical and finite element modeling provide tools to guide the sensor structure design and predict strain sensing responses. These miniaturized sensors are well suited for accurate measurement of local deformation of target objects using a simple stick-on process, as demonstrated by sensing multimodal deformations of a soft continuum arm. The combination of large stretchability, small hysteresis, fast response speed, directional strain response, and small sensor footprint is critical for accurately measuring local strain of large, complex, and multimodal deformations, as found in animals (e.g., octopus arms and elephant trunks), humans (e.g., lungs), and soft robots. The scalable fabrication process and predictable sensor performance further expand opportunities for practical implementation. Future work may involve 3D electrode designs for increased strain range and gauge factor, as well as enhanced resistance to normal pressure and electromagnetic interference. Our findings suggest potential applications for accurately measuring large and complex deformations of soft bodies in wearable and implantable devices, soft robot proprioception, and human-machine interfaces.

## MATERIALS AND METHODS

### Fabrication of 2D precursors

The fabrication of the origami-inspired 3D electrodes began with spin coating a sacrificial layer of polymethyl methacrylate (PMMA) on a glass slide at 3000 rpm, followed by deposition of a parylene C layer (5 μm in thickness) as the first functional layer. Deposition, patterning, and etching of a metal layer (Cr/Au, 25 nm/200 nm in thickness) defined the electrode area and the interconnects. Deposition of another parylene C layer (5 μm in thickness) formed the top encapsulation layer. A lithographic patterning step and subsequent etching using oxygen plasma (March RIE) defined the outline of the 2D precursor and exposed the metal contact pads for electrical connection. Deposition and patterning of a thick SU-8 layer (40 μm in thickness) created thickness variation in the 2D precursors with thick electrode plates and thin creases. Undercutting the PMMA sacrificial layer in acetone facilitated the transfer of the 2D precursor from the glass slide to a water-soluble tape [polyvinyl alcohol (PVA)]. Deposition of Ti/SiO_2_ (10 nm/50 nm in thickness) on the back side of the 2D precursor through a shadow mask defined the bonding sites.

### Mechanically guided assembly of 3D electrodes

A strip of silicone elastomer (Ecoflex 00-31, 1 mm in thickness) served as the substrate for mechanically guided assembly. Prestretching the silicone elastomer strip up to 400% using a custom motorized stretcher and treating it with a corona treater (BD-20AC, ETP Inc.) for 30 s created hydroxyl groups on the surface. The same treatment to the SiO_2_ on the 2D precursor’s bonding sites followed by laminating the 2D precursor/PVA tape on the prestretched silicone strip and heating (70°C for 12 min) created strong covalent bonding at the bonding sites. Dissolving the PVA tape with water and releasing the prestretched strip initiated the mechanical buckling of the 2D precursors into 3D non-parallel-plate configuration.

### Sensor encapsulation and electrical connection

The process started with 3D printing a plastic mold, followed by a molding process to create a silicone (Ecoflex 0031) cover layer including a compartment and a microfluidic channel. Treating the surfaces of this top cover layer and the silicone strip containing the 3D electrodes with oxygen plasma (March RIE) followed by mechanical bonding and heating (70°C for 10 min) led to strong bonding. Electrical connection relied on the connection of the exposed metal contact pads to thin enameled copper wires (50 μm in diameter, Remington Industries) with the use of a silver conductive epoxy. The use of coaxial cables (64 μm in diameter, AlphaWire) with sleeves connected to the ground provided enhanced electromagnetic shielding. Injection of liquid glycerol (99+%, Thermo Fisher Scientific) into the compartment and the channel through the two ends assisted by vacuum and sealing of the ends with silicone (Ecoflex 0031) completed the sensor fabrication.

### Tensile tests and data acquisition

A tensile tester (ESM 303, Mark-10) and LCR meter (LCR 6100, GW Instek) served as the mechanical stretching and data acquisition tools to measure strain and capacitance of the sensor. The LCR meter was set at the parallel-capacitance mode with 20-kHz, 2-V peak-to-peak sinusoidal excitation wave. Setting a medium sampling speed with averaging filter on the LCR meter and applying electrostatic discharge mat over the entire testing area helped reduce electrical noise. A custom program synchronized and recorded both capacitance reading and gripper position at a sampling rate of 7 S/s. The use of capacitance-to-digital converters (AD7746, Analog Devices) allowed simultaneous multichannel capacitance measurement. All capacitance data presented in this work are directly measured from the LCR meter or the capacitance-to-digital converters without post-processing.

### Characterization of strain sensing hysteresis

The following definition of DH is used to evaluate most of the hysteresis reported in this work:

$$\mathrm{DH}=\frac{A_{\mathrm{loading}}-A_{\mathrm{unloading}}}{A_{\mathrm{loading}}}\times 100\%$$

Here, *A*_loading_ and *A*_unloading_ are the areas under the sensor’s capacitance change versus strain curve during loading and unloading, respectively, and DH is their percentage difference. This formula applies to strain sensing curves when there is no overlap between the loading and unloading curves (fig. S9). In Fig. 4D, capacitance change curves during loading and unloading can overlap at fast strain rates, resulting in near-zero DH values. In this case, the following hysteresis expression is used for accurate characterization of hysteresis (*47*):

$$\gamma_{H}=\frac{\Delta H_{\max}}{Y_{\mathrm{FS}}}\times 100\%$$

Here, γ_H_ is the hysteresis error, ∆*H*_max_ is the maximum difference between the loading and unloading signal output of the sensor, and *Y*_FS_ is the maximum value of the sensor’s characteristic curve.

### Measurement of sensor response time

A double-acting air cylinder mounted to a breadboard served as the stretching device. A five-way pneumatic solenoid valve (Tailonz Pneumatic) connected to the air cylinder controlled the cylinder motion. A one-channel relay module switch (HiLetgo) controlled the switching action of the solenoid valve via Arduino programming. Two grippers were used to clamp both ends of the sensor with one gripper attached to the threaded stroke of the air cylinder and the other fixed. A capacitance-to-digital converter (AD7746) measured the capacitance at a sampling rate of 90.9 S/s.

### Methods for testing long-term sensor performance

The sensor was fixed by the grippers on the tensile tester (Mark-10) for 15 days. Each day, the tensile tester stretched the sensor to 100% strain for 40 to 45 cycles at a strain rate of 10% s^−1^ with simultaneous capacitance measurement by the LCR meter. The sensor was maintained at 100% strain for 15 s before returning to the unstretched state.

### Methods for testing sensor response to normal pressure

Testing the sensor’s response to normal pressure used a Mark-10 force gauge with a flat header moving at ~0.2 mm/s and pressing the sensor’s sensing region. Such compression was repeated five times for each pressure level and strain level. The capacitance was measured before and during each compression.

### Methods for testing sensor’s robustness against collision and abrasion

The evaluations of sensor’s robustness included a normal stress test and shear stress test. In the normal stress test, the press head speed was set at 20 mm/s to represent a collision scenario (movie S2), and the capacitance was measured before and after applying the normal stress. Setting the stop positions resulted in different normal pressures on the sensor. To test the sensor’s response to shear stresses, a rectangular bar with a tape was attached on a Mark-10 force gauge and moved across the sensor surface. The force gauge, actuated by a linear stage, pushed the bar to move back and forth in the sensor’s longitudinal and transverse directions to create shear stresses. Setting the stop positions resulted in different shear stresses on the sensor. The sensor performance was evaluated by stretching to 100% strain before and after three consecutive normal stress or shear stress tests.

### Fabrication of stretchable electromagnetic shielding layers

The preparation of a shielding layer started with laser ablation of a sheet (0.5 mm in thickness) of stretchable silver-plated knitted fabrics (Shieldex P130, V Technical Textiles Inc.) with slit patterns. Attaching two patterned sheets on the top and bottom surfaces of the encapsulated sensors followed by spin coating thin layers of silicone elastomer (Ecoflex, 0.2 mm in thickness) fixed the shielding layers. Micro-coaxial cables (9442, AlphaWire) provided electrical connection to the shielded sensor: The core connected the sensor’s contact pad to the capacitance reading terminal of the measurement equipment, and the shield connected the equipment’s ground terminal to provide proper grounding.

### Fabrication and strain sensing of soft continuum arm

A rectangular pillar (dimension: 50 × 50 × 150 mm^3^) made of Ecoflex 00-31 served as a soft continuum arm for proof-of-concept demonstrations. Six sensors (strip length, 15 mm; electrode *L* = 500 μm) were separated into two rosette patterns and attached to two adjacent faces (*S*_1_ and *S*_2_) on the arm with the application of a small amount of Ecoflex 00-31 as adhesive. Deformation of the arm was realized by manual loading, followed by holding in the final loaded shape for 20 s, and returned to the undeformed shape. Three capacitance-to-digital converters (AD7746 boards) recorded the capacitance changes from all the sensors at a sampling rate of 3 S/s.

### Finite element analysis

Simulations of the origami-inspired strain sensors were performed by commercial software ABAQUS (for mechanics simulation) and COMSOL (for electrostatics simulation). To simulate the mechanical deformations of the 3D electrodes and the serpentine interconnects, four-node shell elements (S4R) and composite layup were used to define and mesh the structures. Fine mesh was used (~23,000 elements for mechanically guided assembly and ~16,000 elements for stretching of serpentine interconnects) to ensure accuracy, and geometric nonlinearity was enabled for simulating large deformations. For simulating the deformation of sensors attached to silicone elastomer substrates, four-node linear tetrahedron solid elements (C3D4) with fine mesh (~210,000 elements) were used. The Young’s moduli of parylene C, SU-8, and Au are *E*_Pa_= 2.8 GPa, *E*_SU_= 4.02 GPa, and *E*_Au_= 78 GPa, respectively. The Poisson’s ratios are ν_Pa_ = 0.4, ν_SU_ = 0.22, and ν_Au_ = 0.44, respectively. The yield strain of ebeam evaporated Au thin film is 0.7% (*58*). The mechanical properties of Ecoflex are captured by the following Mooney-Rivlin strain potential constitutive model

$$U=C_{10}(\bar{I_{1}}-3)+C_{01}(\bar{I_{2}}-3)+D_{1}^{-1}{\left(J^{\mathrm{el}}-1\right)}^{2}$$

where $\bar{I_{1}}$ and $\bar{I_{2}}$ are the first and second invariants of the left Cauchy-Green deformation tensor, respectively, and *J*^el^ is the elastic volume ratio representing the thermal expansion.

The capacitance output of the sensor was simulated in COMSOL Multiphysics. The structures of the 3D electrodes with different folding angles were imported into COMSOL Multiphysics and meshed by four-node linear tetrahedron solid elements (C3D4) with finer size than the electrode thickness (~820,000 elements). One plate of the capacitor was charged for 1 V, and the other was grounded. The electrical potential was normalized to the bias voltage (1 V). The capacitance value was obtained by global evaluation. The dielectric constants used are 46.8, 2.95, and 2.8 for glycerol, parylene C, and Ecoflex, respectively.

## Supplementary Materials

### This PDF file includes:

Notes S1 to S4 Figs. S1 to S26 Tables S1 to S3 Legends for movies S1 and S2 References
