# Supplementary material for: High-stretchability and low-hysteresis strain sensors using origami-inspired 3D mesostructures
Source: Sci Adv. 2023 Aug 25;9(34):eadh9799. doi: 10.1126/sciadv.adh9799 (PMC10456843; doi:10.1126/sciadv.adh9799)
Supplement: Supplementary file 1 — Notes S1 to S4 Figs. S1 to S26 Tables S1 to S3 Legends for movies S1 and S2 References [file sciadv.adh9799_sm.pdf]

Science Advances
AAAS

Supplementary Materials for

High-stretchability and low-hysteresis strain sensors using origami-inspired 3D mesostructures

Xinghao Huang *et al.*

Corresponding author: Hangbo Zhao, hangbozh@usc.edu

Sci. Adv. **9**, eadh9799 (2023)
DOI: 10.1126/sciadv.adh9799

The PDF file includes:

Note S1 to S4
Figs. S1 to S26
Tables S1 to S3
Legends for movies S1 and S2
References

Other Supplementary Material for this manuscript includes the following:

Movies S1 and S2

Supplementary Note S1. Analytical modeling of the capacitance of the origami-inspired 3D electrodes.

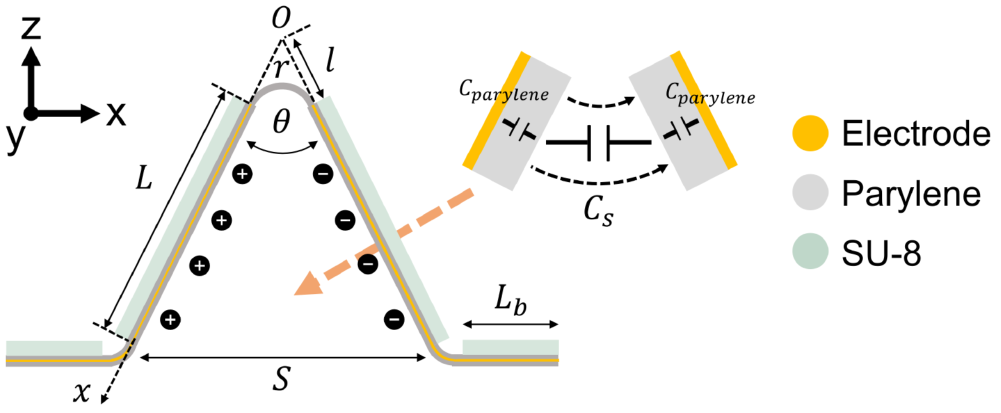

Here is a list of parameters used in the analytical modeling:
L: electrode length
L_b: bonding side length
W: electrode width (into the page)
W_b: bonding site width (into the page)
$t_{parylene}$: parylene layer thickness
S: distance between the two bonding sites at the bottom of the two electrodes
r: length of the parylene arc that connects the two electrodes at the center
l: distance from the electrode end to the angle's intersection point
θ: angle between the two electrodes
$C_{parylene}$: capacitance across the parylene layer
C_s: glycerol capacitance formed at the overlapping regions between the two electrodes
C_{in}: total inner capacitance formed at the overlapping regions between the two electrodes
C_{xy}: fringe capacitance at the *xy* plane along the edge of the electrodes
C_{xz}: fringe capacitance at the *xz* plane, mainly at the top and bottom of the electrodes
V: applied potential difference
σ: charge per unit area
ε_0: permittivity of free space
$\varepsilon_{glycerol}$: dielectric constant (relative permittivity) of liquid glycerol
$\varepsilon_{ecoflex}$: dielectric constant (relative permittivity) of Ecoflex elastomer

<u>Kinematic constraints</u>
The angle θ is defined by L, S, and r. Assuming the arc r is tangent to the electrodes, there is a geometric relationship:

$$\theta = 2\sin^{-1}\left(\frac{\frac{1}{2}S}{l + L}\right)$$

where l can be calculated as

$$l = \frac{\tan\left(\frac{\pi}{2} - \frac{1}{2}\theta\right)}{\left(\frac{\pi}{2} - \frac{1}{2}\theta\right)} \cdot \frac{r}{2}$$

Since the only unknown is θ, we can find the angle θ given the distance between the bonding sites S.

<u>Estimation of total capacitance</u>

Assuming that the electric field lines between the two folded electrodes have arc shapes (*59*), the charge per unit area on these two folded electrodes can be expressed as:

$$\sigma = \frac{\varepsilon V}{\theta x}$$

Using the charge-voltage relationship, all charges can be summed up along the edges of the electrodes:

$$C_s = \frac{q}{V} = \frac{1}{V}\int_{r_1}^{r_2} \sigma dA = \int_{r_1}^{r_2} \frac{\varepsilon w}{\theta x} dx$$

Here, r_2 *and* r_1 are the arc lengths from point O to the two ends of the electrodes, which are $l + L$ and l, respectively. Since the space between the two electrodes is filled with liquid glycerol, $\varepsilon_{glycerol}$ is used as the dielectric constant. Integrating the equation above will give:

$$C_s = \frac{\varepsilon_0 \varepsilon_{glycerol} w}{\theta} \ln\left(\frac{r_2}{r_1}\right) = \frac{\varepsilon_0 \varepsilon_{glycerol} w}{\theta} \ln\left(\frac{l + L}{l}\right)$$

Since the electrodes are insulated by parylene C, the parylene layer's capacitance $C_{parylene}$ and the glycerol capacitance C_s will form a series of capacitors as shown in the schematic drawing above. Therefore, the total inner capacitance C_{in} in the space between the two electrodes is:

$$\frac{1}{C_{in}} = \frac{1}{C_{parylene}} + \frac{1}{C_s} + \frac{1}{C_{parylene}}$$

$$C_{in} = \frac{C_{parylene} C_s}{2C_s + C_{parylene}}$$

It is worth noting that the use of liquid glycerol as the dielectric medium can possibly generate electrical double layer capacitance. However, the extremely low ionic conductivity of the glycerol used in the sensors could only result in double layer capacitance that is several orders of magnitude higher than C_{in} (2-3 pF) (*60*). Since this possible double layer capacitor $C_{parylene}$ and glycerol capacitance C_s are connected in series, its high capacitance will have little influence on the total capacitance C_{in}.

Modeling of the fringe capacitance is adopted from previous studies (*25, 26, 42*) that use conformal mapping to transform the non-parallel-plate capacitance to parallel-plate capacitance in complex domains. According to the geometry presented in Fig. 1A, the capacitance C_{xz} is as follows:

$$C_{xz} = 2w\varepsilon_0\varepsilon_{glycerol}\left(\frac{\ln\left(\frac{2\pi}{\theta}\right)}{2\pi-\theta}\right) + 2w\varepsilon_0\varepsilon_{ecoflex}\left(\frac{\ln\left(\frac{2\pi}{2\pi-\theta}\right)}{\theta}\right)$$
$$+\frac{2\varepsilon_0\varepsilon_{ecoflex}w_b}{\pi}\ln\left(\frac{2L_b+S}{S}+\sqrt{\left[\frac{(2L_b+S)}{S}\right]^2-1}\right)$$

The first term represents the fringe capacitance at the top of the angled electrodes, where the electric field exists within the liquid glycerol. The second term represents the fringe capacitance at the bottom of the angled electrodes, while the third term represents the fringe capacitance generated by the two flat plates at the bonding sites. Both fringe capacitances exist mostly inside the bottom Ecoflex substrate. The expression for the fringe capacitance C_{xy} is shown below, which is the capacitance generated between the edges of the angled electrodes:

$$C_{xy} = \int_{l}^{l+L}\frac{\varepsilon_0\varepsilon_{glycerol}}{\pi}\ln\left(\frac{l+L}{l}\left(1+\ln\left(1+\frac{2\pi w}{\theta x}+\ln\left(1+\frac{2\pi w}{\theta x}\right)\right)\right)\right)dx$$

Since C_{in}, C_{xy}, and C_{xz} are connected in parallel, the total capacitance can be estimated as:

$$C_{total} \approx C_{in} + C_{xy} + C_{xz}$$

Explanation for discrepancies between models and experimental results for sensors with long electrodes

In Fig. S8B, the capacitance reductions from the analytical solution and simulation are smaller than those in the experiments. These discrepancies may result from several factors. First, both the analytical solution and simulation account for the Ecoflex at the bottom of the electrodes and exclude the Ecoflex surrounding the 3D electrodes in the top cover. Under large stretching, the reduced gap between the Ecoflex cover and 3D electrodes leads to decreases in C_{xy} and C_{xz} due to a lower dielectric constant of Ecoflex compared to glycerol. This effect is more pronounced in sensors with longer electrode lengths than those with shorter lengths. Other factors such as slight tilting of bonding sites due to stress concentration and the presence of the slit at the center crease of the 3D electrodes (not considered in analytical modeling) can also contribute to discrepancies observed between analytical modeling, simulation, and experiments.

Supplementary Note S2: Influence of normal pressure on strain sensing.

Fig. S18 shows the capacitance responses of a representative sensor to uniaxial stretching under coexisting uniaxial stretching and normal pressure. The capacitance is normalized to the initial capacitance before stretching and without normal pressure. The error bars on each data point indicate the standard deviation of capacitance changes across 5 repeated tests. An increase in normal pressure from 0 to 30 kPa leads to partial unfolding of the 3D electrodes, resulting in a capacitance decrease. This reduction is 4.3% for unstretched sensor at 30 kPa. When the sensor is stretched under 30% strain, the strain-induced relative capacitance change is -12.1%, while the normal pressure-induced relative capacitance change is -3.2%. The influence of normal pressure on strain sensing is even smaller at 60% strain, as the 3D electrodes further unfold. The relative capacitance change contributed by stretching and normal pressure is -21.1% and -3.9%, respectively. The results suggest that the influence of normal pressure from 0 to 30 kPa on strain sensing decreases as the strain increases from 0 to 60%, and it is expected to be even smaller for large strain sensing. It should be noted that the sensor is only sensitive to normal pressure applied above its active sensing area (< 5 mm^2). Modification of the sensor design can further reduce its sensitivity to normal pressure, including the use of top silicone cover with larger thickness and higher modulus.

Supplementary Note S3: Characterization of sensors' mechanical robustness against collisions and abrasions.

Mechanical robustness against collisions and abrasions is crucial for strain sensors used in wearable applications. Movie S2 shows the sensor's mechanical robustness tests. To characterize the sensor performance against collisions, dynamic compression tests are performed utilizing a fast-actuating press head that moves at 20 mm/s to apply normal pressure at the sensor's sensing region (Fig. 19A). The sensor performance is evaluated by measuring the sensor capacitance before and after compression. Fig. S19B shows that the sensor can still be stretched to 100% strain after compression with 240 kPa peak pressure above the sensing area. The degree of hysteresis remains mostly around 2% for all applied pressure levels. No significant changes on 3D electrodes are observed until applying a compression with 240 kPa peak pressure, where the 3D electrodes significantly tilt to one side (Fig. S19C). The gauge factor changes from -0.22 at the initial state to -0.26 after 240 kPa normal pressure, which may be explained by the gradual tilting of the electrodes after repeated compression tests. Further increasing the pressure (~360 kPa) snaps the serpentine interconnects and breaks the 3D electrodes within the Ecoflex compartment. Modifications including a more damped, pressure-resistant cover may better attenuate the dynamic normal pressure from collisions.

To characterize the sensor performance against abrasions, both longitudinal and transverse shear stresses are applied on two sensors. Fig. S19D shows that the sensor can function after an applied shear stress of 46 kPa along the sensor's longitudinal direction, despite misalignment in the serpentine interconnects (Fig. S19E). Similar misalignment in the serpentine interconnects also occurs when the sensor is under 40 kPa shear stress in the transverse direction (Fig. S19G). Shear in the transverse direction can also undermine the bonding strength at the 3D electrodes' bonding

sites, resulting in increased hysteresis (Fig. S19F). Overall, the sensors can maintain their performance at 22~23 kPa shear stress in both the longitudinal and transverse directions without obvious degradation. Larger shear stresses can cause damage to the serpentine interconnects and lead to the delamination of the 3D electrodes from the bonded surface. Shear-resistant designs of liquid channels and serpentine structures may help increase the sensor's resistance to shear damage.

Supplementary Note S4: Machine learning for classifying deformation modes.

To classify single deformation modes for the continuum arm, 1,119 sets of relative capacitance changes are collected during multiple trials of 9 deformation modes, including bending (4 directions), twisting (2 directions), elongation, compression, and no deformation. Each data point includes the capacitance changes from the 6 sensors attached on the arm. After each data point is labeled with its corresponding deformation, the entire dataset is randomly split, with 70% used for training and 30% for testing. K-nearest neighbor (KNN), Linear Support Vector Classifier (SVC), and Random Forest Classifier (RFC) with default parameters from Scikit-Learn 1.2.2 are chosen as the machine learning algorithms for deformation prediction. The accuracies for classifying deformation modes using the trained KNN, SVC, and RFC classification models on the test dataset are 99.7%, 99.1%, and 99.7%, respectively. The high accuracies are explained by the highly distinguishable features of the 9 different deformation modes (Fig. S26). More datasets and other machine learning algorithms including deep learning may be needed for recognition of more complex deformations such as various hybrid deformations.

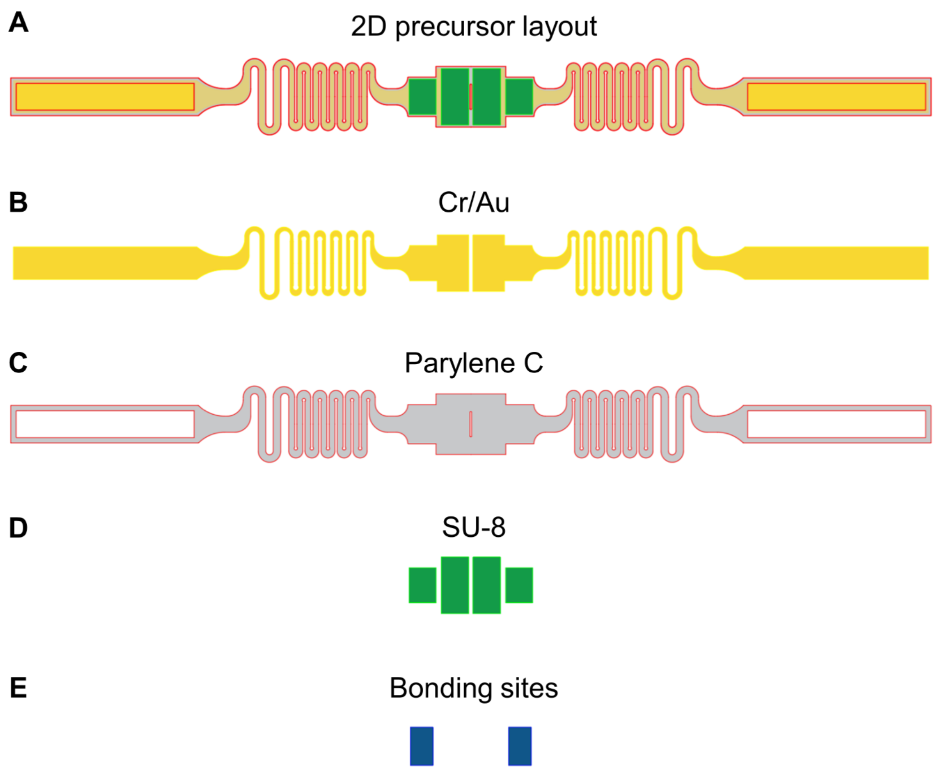

Fig. S1. Layouts of the 2D precursor of the basic origami-inspired capacitive strain sensor design. (**A**) Overview of the stacked 2D precursor layouts. (**B**) Layout of the Cr/Au metal layer. (**C**) Layout of the parylene C layer. (**D**) Layout of the SU-8 stiffener layer. (**E**) Layout of the bonding sites on the backside of the 2D precursor.

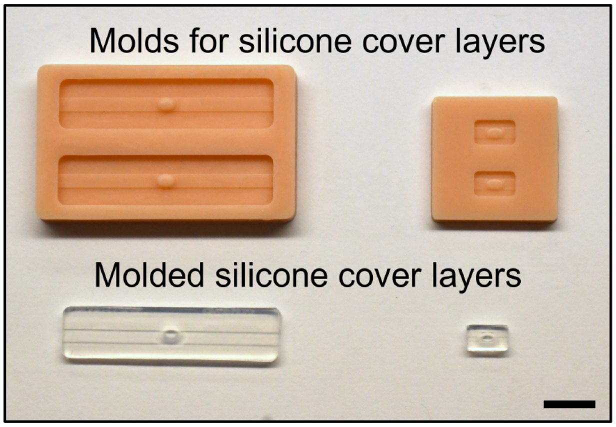

Fig. S2. Optical images of 3D printed molds and the corresponding molded silicone top encapsulation covers for two sensor designs with different electrical interconnect lengths. Scale bar, 1 cm.

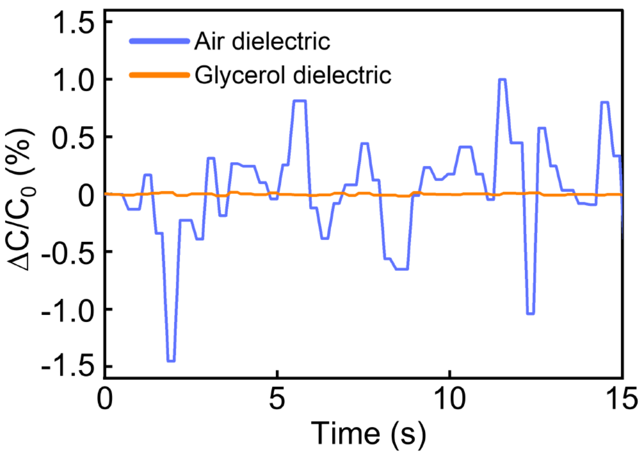

Fig. S3. Comparison between the noise levels of two sensors (*L* = 1.0 mm, trapezoidal electrodes) using air and glycerol as the dielectric material. Capacitance values for both sensors are recorded by LCR-6100 at "LOW Speed" option when the sensors remain stationary.

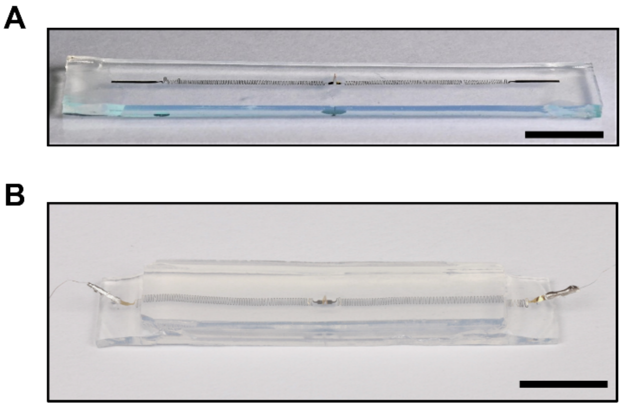

Fig. S4. Optical images of the sensor with long serpentine interconnects. (**A**) Before encapsulation of the electrodes and (**B**) after encapsulation filled with liquid glycerol. Scale bars, 1 cm.

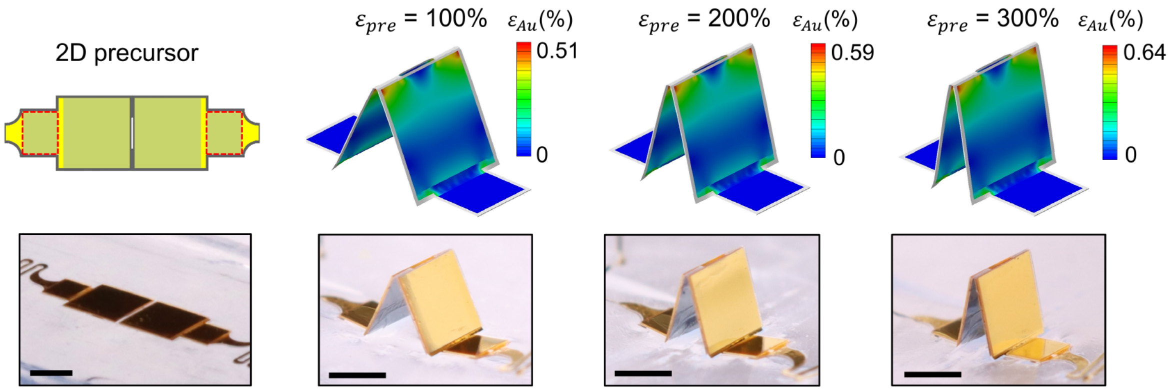

Fig. S5. FEA and experimental images of an origami-inspired 3D non-parallel-plate capacitor with different folding angles formed by compressive buckling with different prestrain. The regions on the 2D precursor encircled by red dashed lines indicate bonding sites. The contours in the FEA images indicate the strain in the gold layer. Scale bars, 1 mm.

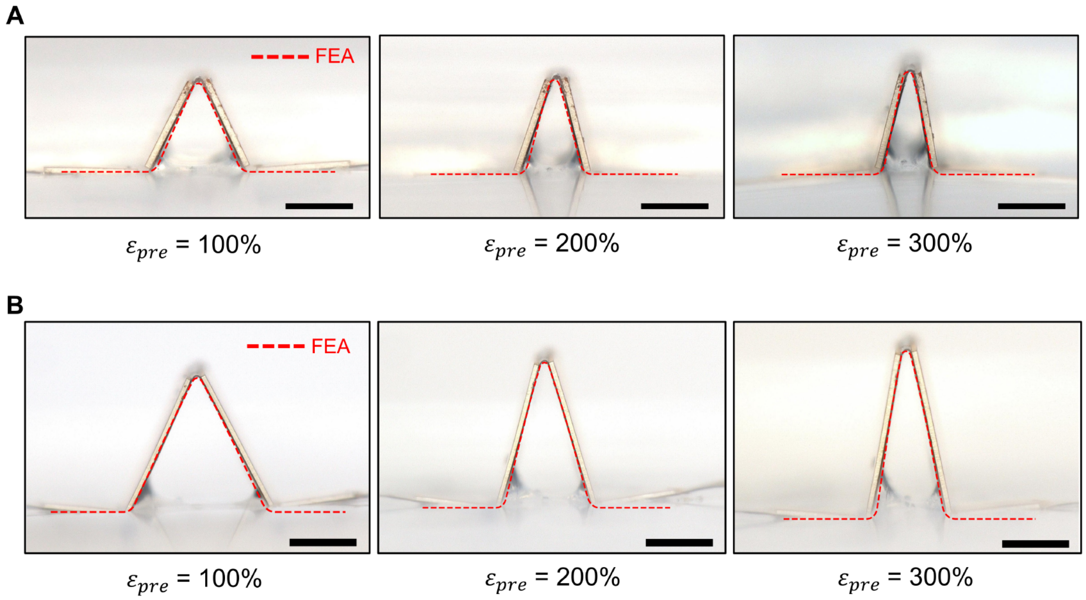

Fig. S6. Sideview optical images and the corresponding simulated profiles of the origami-inspired 3D non-parallel-plate capacitor with different prestrain. (**A**) 0.5 mm electrode length and (**B**) 1 mm electrode length. Scale bars, 250 µm in A and 500 µm in B.

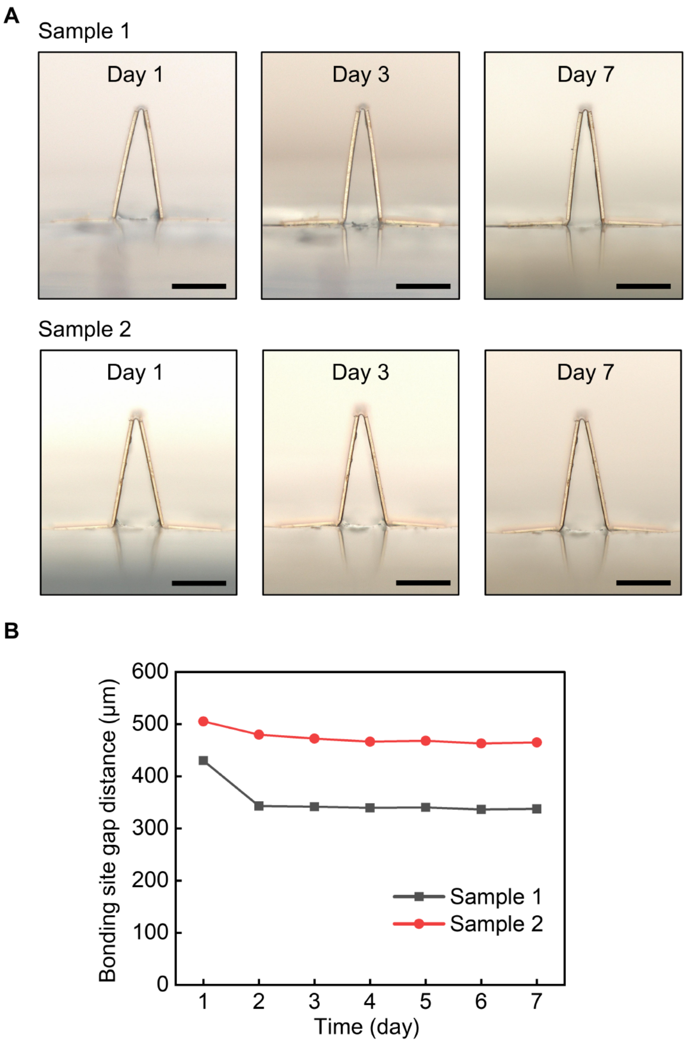

Fig. S7. Characterization of structural changes of the 3D electrodes over a week. (**A**) Sideview images of two 3D electrodes on day 1, day 3, and day 7 after the mechanically guided assembly process. (**B**) Distances between the two bonding sites measured from day 1 to day 7. Scale bars, 500 μm.

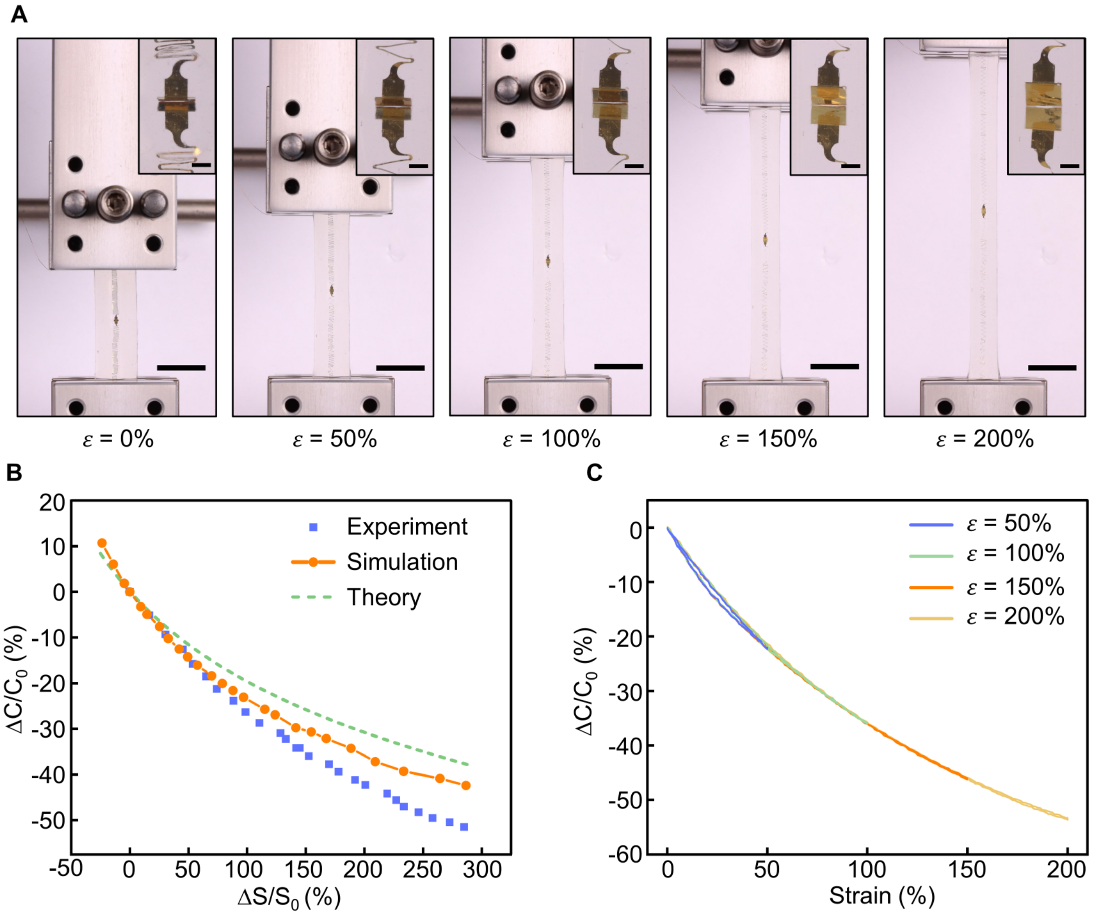

Fig. S8. Characterization of a strain sensor with 1 mm electrode length. (**A**) Optical images of a sensor under uniaxial stretching at 0%, 50%, 100%, 150%, and 200%, with insets showing close-up views of the 3D electrodes. (**B**) Simulation, analytical, and experimental results for the relative change of bonding site distance and the corresponding relative capacitance change. The sensor has an electrode length of L = 1 mm and width of W = 1 mm. C_0 corresponds to the capacitance with a bonding site distance of S_0 = 403 µm. (**C**) Relative change in capacitance of the sensor, with different prestrain, during loading and unloading at applied strains of 50%, 100%, 150%, and 200%. Scale bars, 1 cm in A (500 µm in insets).

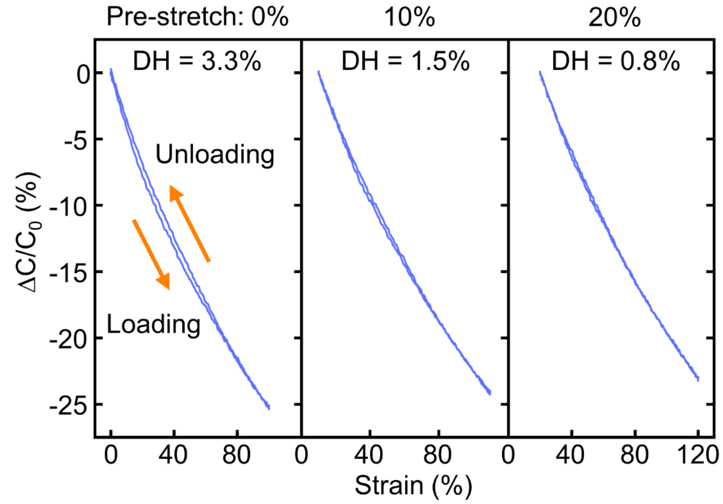

Fig. S9. Relative capacitance change of a representative sensor under 100% applied strain with 0%, 10%, and 20% pre-stretch. The DH values are 3.3%, 1.5%, and 0.8%, respectively.

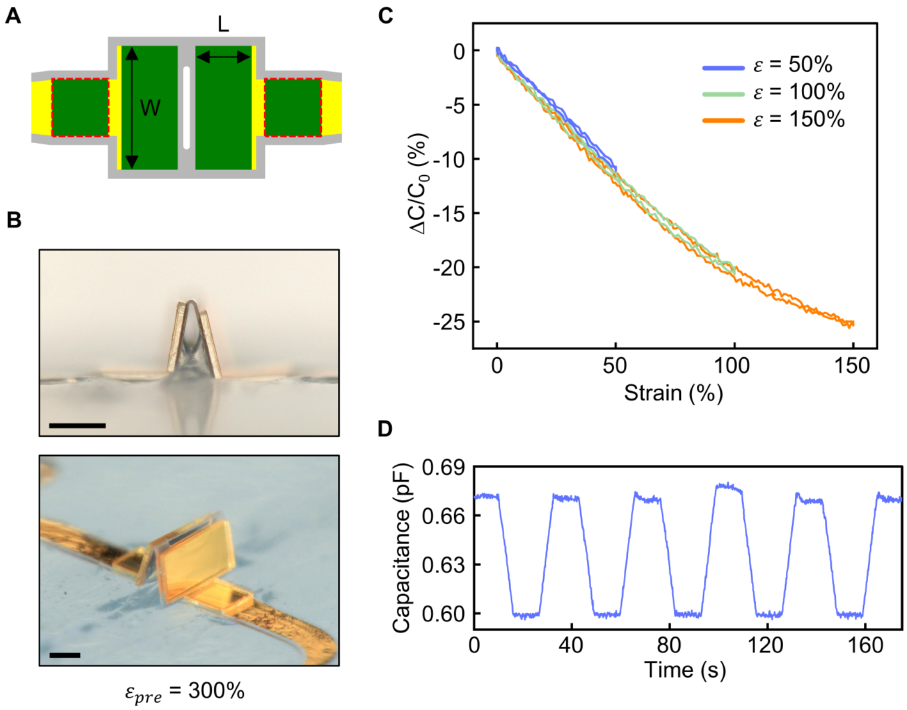

Fig. S10. Design and characterization of a 3D capacitive strain sensor with electrode size of *L* = 250 µm and *W* = 550 µm. (**A**) 2D layout of the electrode design. The regions encircled by red dashed lines represent bonding sites (area: 250 µm × 250 µm). (**B**) Optical images of the front and angled view of the 3D electrodes formed by compressive buckling with 300% prestrain. (**C**) Relative capacitance change of the sensor during uniaxial loading and unloading at 50%, 100%, and 150% applied strain. (**D**) Capacitance change during cyclic 50% uniaxial loading and unloading. Scale bars, 200 µm in B.

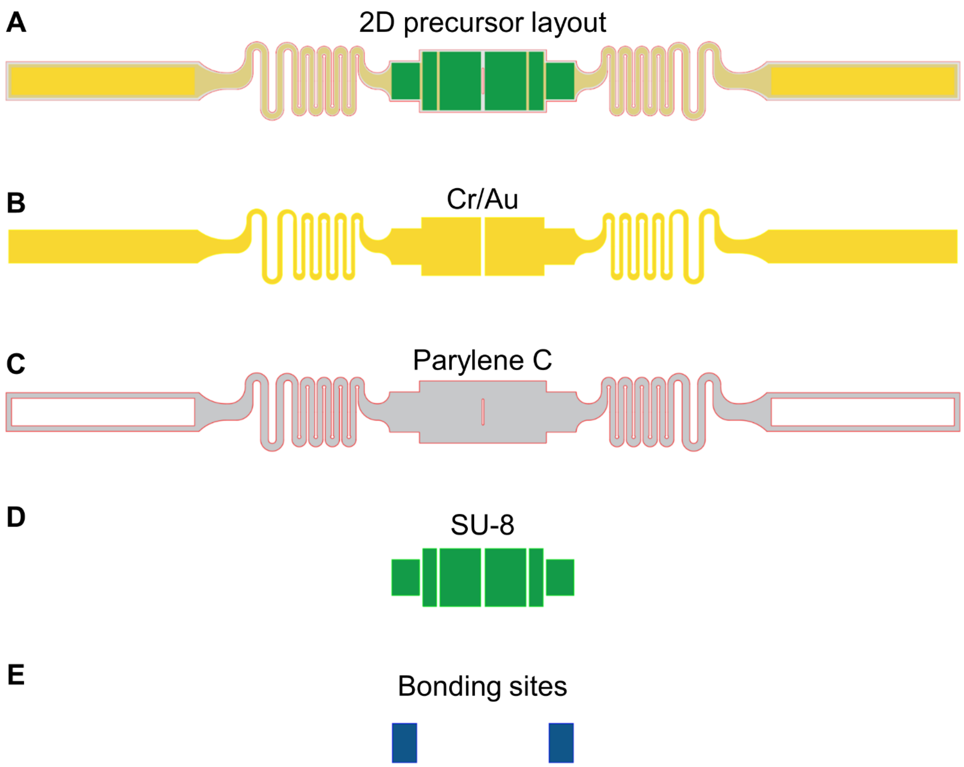

Fig. S11. Layouts of the 2D precursor of the five-crease, origami-inspired capacitive strain sensor design. (**A**) Overview of the stacked 2D precursor layouts. (**B**) Layout of the Cr/Au metal layer. (**C**) Layout of the parylene C layer. (**D**) Layout of the SU-8 stiffener layer. (**E**) Layout of the bonding sites on the backside of the 2D precursor.

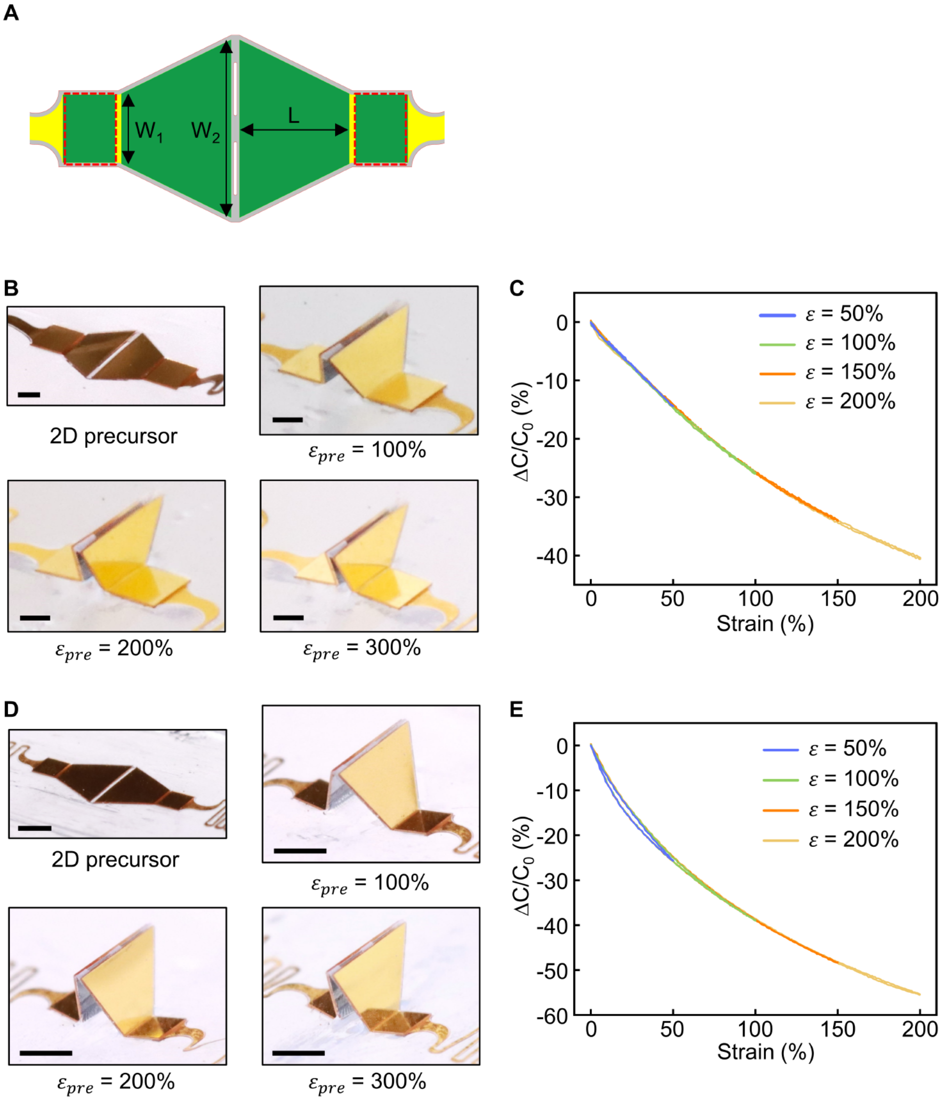

Fig. S12. Design and characterization of 3D capacitive strain sensors based on trapezoid-shape electrodes. (**A**) 2D layout and geometric parameters of the trapezoidal electrode design. The regions encircled by red dashed lines represent bonding sites. (**B**) Optical images of electrodes with L = 0.5 mm formed by compressive buckling with different prestrain. (**C**) Relative capacitance change of a representative sensor based on trapezoidal electrodes (L = 0.5 mm, W_1 = 0.7 mm, W_2 = 1.5 mm, 300% prestrain) during uniaxial loading and unloading at 50%, 100%, 150%, and 200% applied strain. (**D**) Optical images of electrodes with L = 1 mm formed by compressive buckling with different prestrain. (**E**) Relative capacitance change of a representative sensor based on trapezoidal electrodes (L = 1 mm, W_1 = 0.7 mm, W_2 = 1.7 mm, 300% prestrain) during uniaxial loading and unloading at 50%, 100%, 150%, and 200% applied strain. Scale bars, 500 μm in B, 1 mm in D.

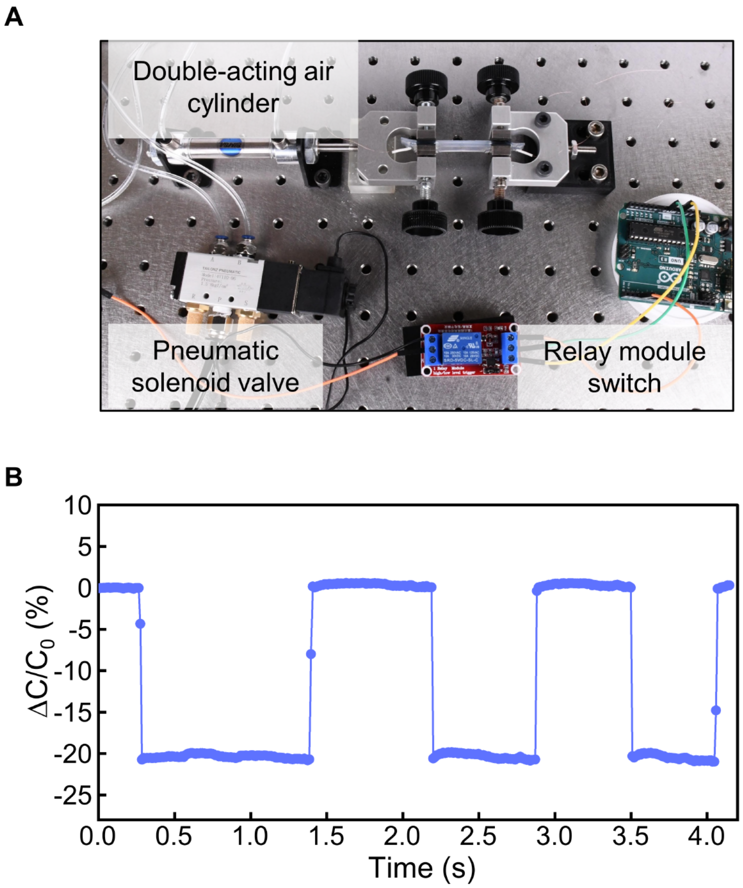

Fig. S13. Measurement of the sensor response and recovery time. (**A**) Experimental setup for measuring the sensor response and recovery time. (**B**) Capacitance responses from a representative sensor (L = 0.5 mm) subjected to 100% stretching and release for multiple cycles at fast speeds.

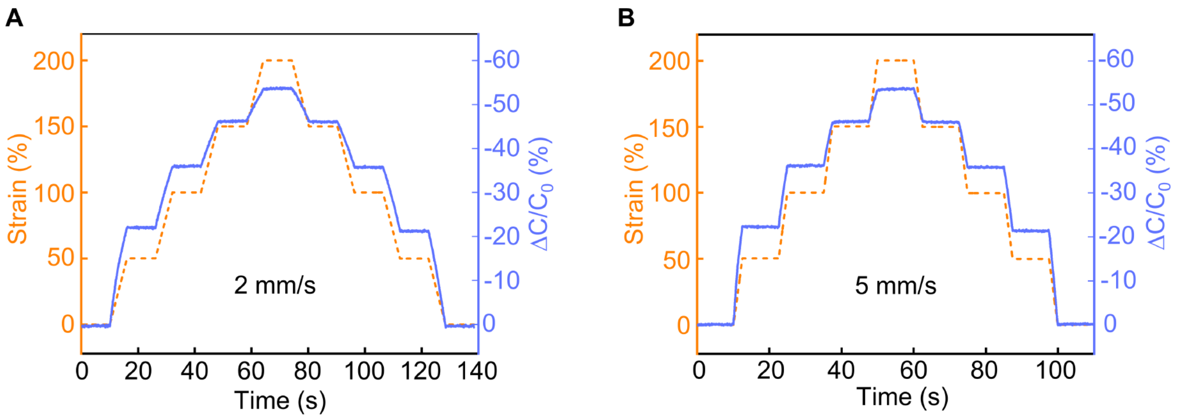

Fig. S14. Capacitance response of a representative sensor (*L* = 1 mm) subjected to a series of step-up strain of 50% to a maximum of 200% followed by step-down strain to the initial state. The stretching and releasing rates are (**A**) 2 mm/s (strain rate: 8.3% s^{-1}) and (**B**) 5 mm/s (strain rate: 20.8% s^{-1}), respectively.

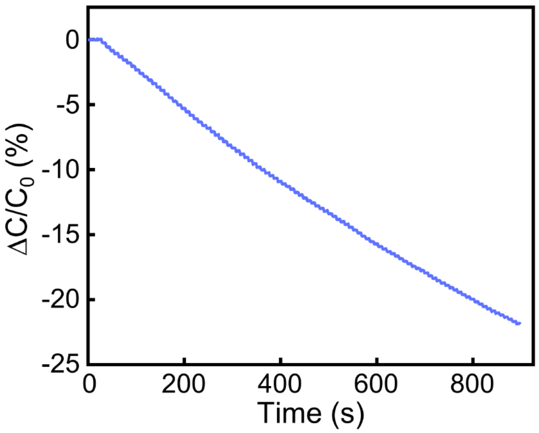

Fig. S15. Relative capacitance change of a representative sensor (L = 0.5 mm) under stretching (up to 100% strain) with fine stepping profile (step size of 1% strain).

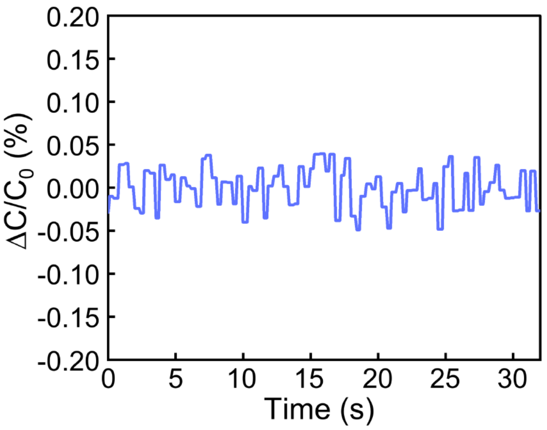

Fig. S16. Representative noise level of a 3D capacitive strain sensor.

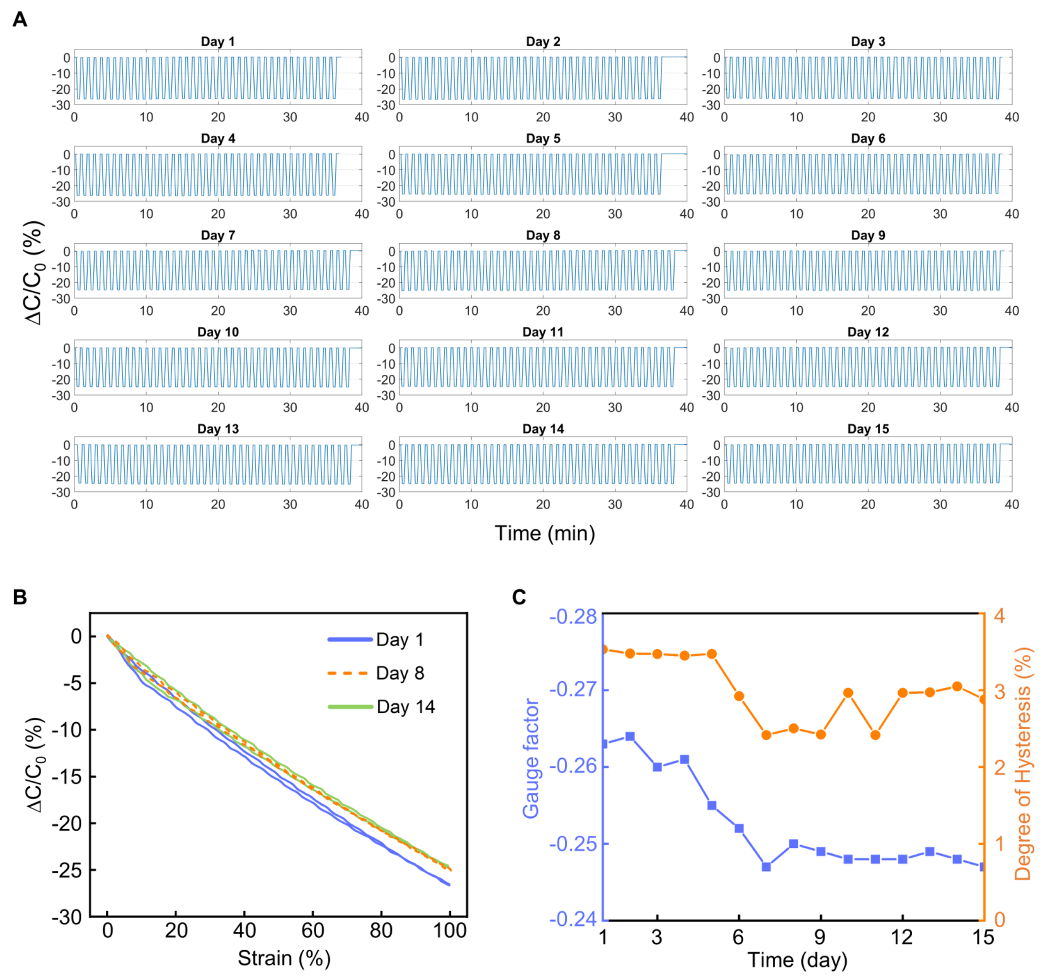

Fig. S17. Test of sensor performance over a 15-day period. (**A**) Capacitance responses of a sensor (L = 0.5 mm, trapezoidal electrode) being stretched to 100% strain for 40~45 cycles a day at a strain rate of 10% s^{-1}. (**B**) Representative capacitance and strain curves on day 1, day 8, and day 14. (**C**) Changes in the gauge factor and the degree of hysteresis from day 1 to day 15.

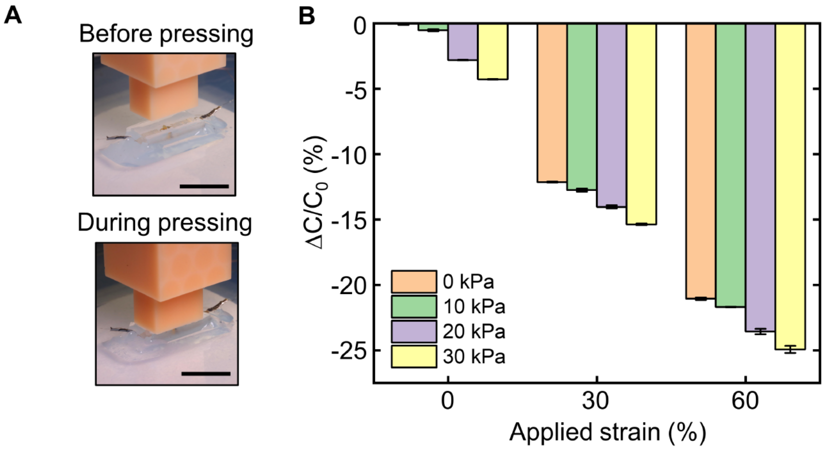

Fig. S18. Sensor responses to normal pressure. (**A**) Optical images of a sensor before and during a static, persistent normal pressure. (**B**) Capacitance response of a sensor stretched to different levels (0%, 30%, and 60%) under different static, persistent normal pressures. Scale bars, 1 cm.

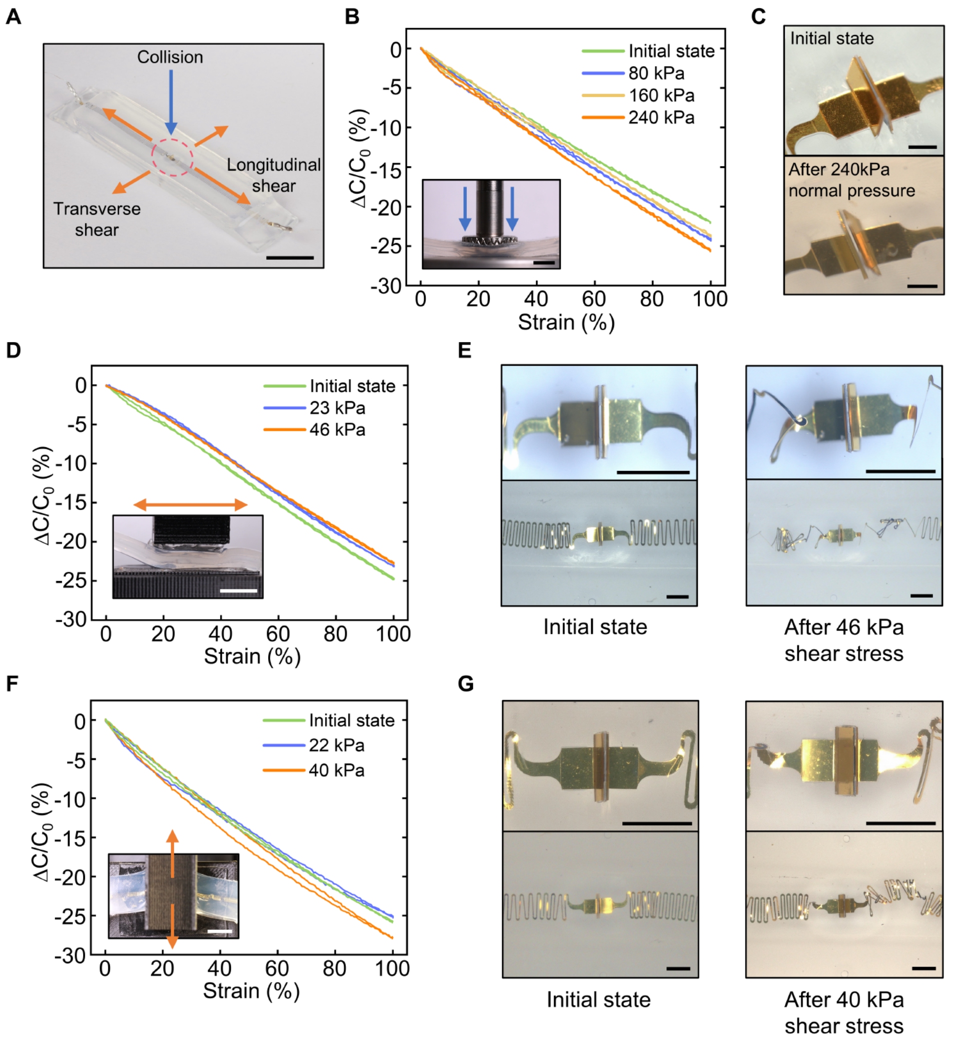

Fig. S19. Characterization of sensors' mechanical robustness against collision and abrasion. (**A**) Loading directions for collision and abrasion tests. (**B**) Relative capacitance changes of the sensors under 100% strain before and after applying peak normal pressures of 80 kPa, 160 kPa, and 240 kPa. (**C**) Optical images of the 3D electrodes in the initial state and after applying a peak normal pressure of 240 kPa. The pressure is applied at a speed of 20 mm/s. (**D**) Relative capacitance changes of the sensors under 100% strain before and after applying peak shear stresses of 23 kPa and 46 kPa in the sensor's longitudinal direction. (**E**) Optical images of the 3D electrodes and serpentine interconnects in the initial state and after applying a peak shear pressure of 46 kPa in the sensor's longitudinal direction. (**F**) Relative capacitance changes of the sensors under 100% strain before and after applying peak shear stress of 22 kPa and 40 kPa in the sensor's transverse direction. (**G**) Optical images of the 3D electrodes and serpentine interconnects in the initial state and after applying a peak shear stress of 40 kPa in the transverse direction. Scale bars, 1 cm in A; 5 cm in B, D, and F; 500 µm in C; 1 mm in E and G.

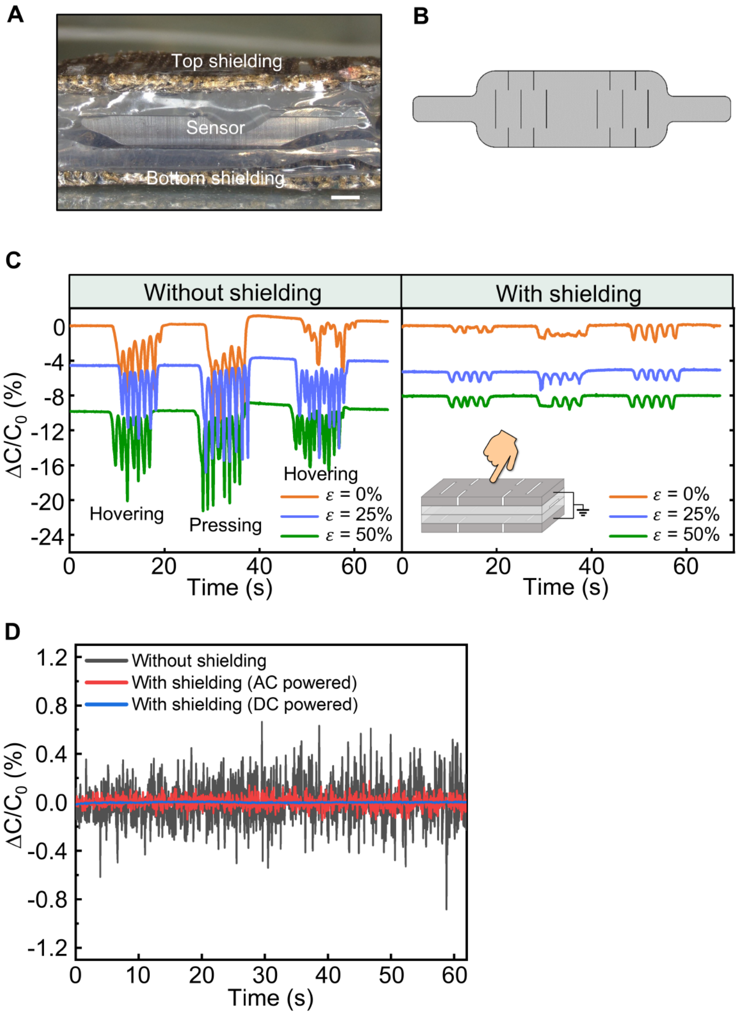

Fig. S20. Design and performance of strain sensors with electromagnetic shielding. (**A**) Optical image (cross-sectional view) of a sensor with two electromagnetic shielding layers on the top and bottom. (**B**) Kirigami-inspired cut design in the electromagnetic shielding layers. (**C**) Comparison of sensor responses without and with electromagnetic shielding layers to proximity to human finger and pressing under different stretching conditions. (**D**) Comparison of noise levels for a sensor with and without the electromagnetic shielding layers. Scale bar, 1 mm.

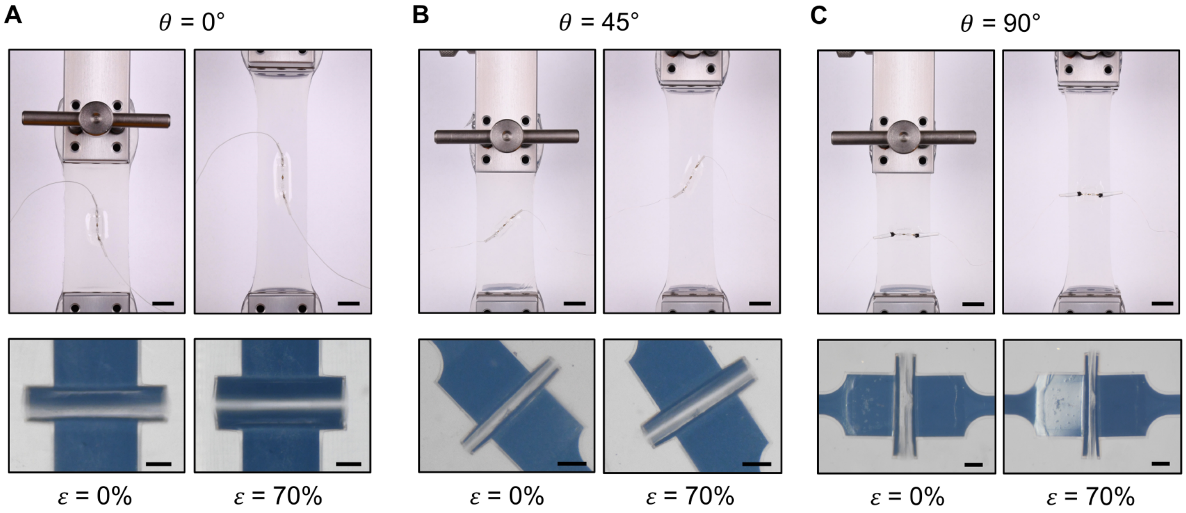

Fig. S21. Optical images of the directional strain sensing test. The sensor is attached to a silicone slab at (**A**) 0°, (**B**) 45°, and (**C**) 90° angles with respect to the longitudinal direction of the silicone slab. For each direction, the slab is stretched from 0% (left) to 70% strain (right). Scale bars, 2 cm for the top row and 250 μm for the bottom row.

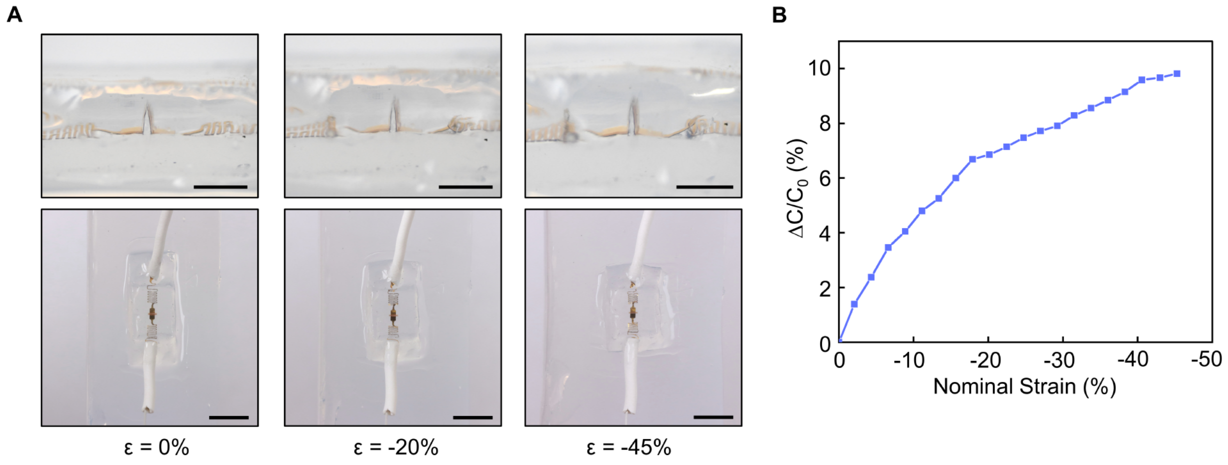

Fig. S22. Changes in the 3D electrodes, serpentine interconnects, and capacitance of the sensor under compressive strain. (**A**) Side view (top row) and top-down view (bottom row) of the sensor under compressive strain of 0%, -20%, and -45%. (**B**) The relative capacitance change of the sensor under different compressive strain. Scale bars, 1 mm for the top row and 5 mm for the bottom row in A.

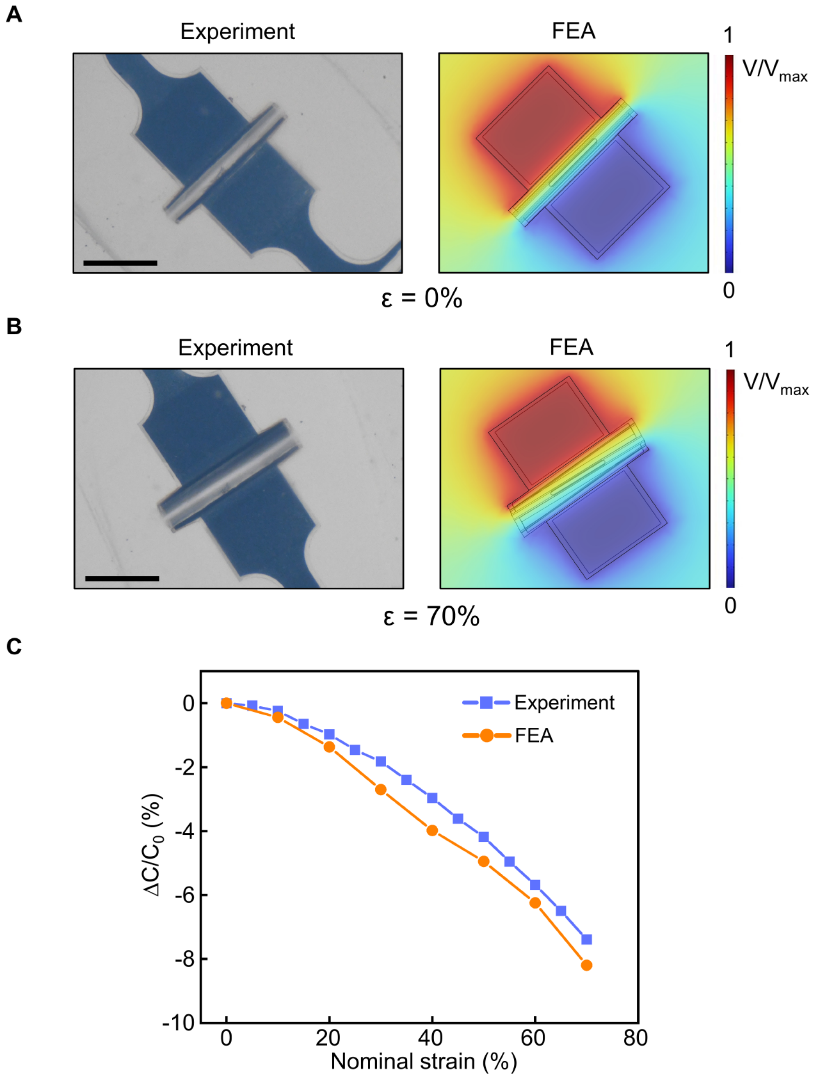

Fig. S23. FEA of sensor stretching at θ = 45° direction. The sensor is attached to a silicone slab at 45° angle with respect to the longitudinal direction of the silicone slab. (**A**) Experimental images and simulated electric potential field for the sensor at 0% strain. (**B**) Experimental images and simulated electric potential field for the sensor being stretched to 70% nominal strain. (**C**) Capacitance change from experiment and simulation. Scale bars, 500 μm in A and B.

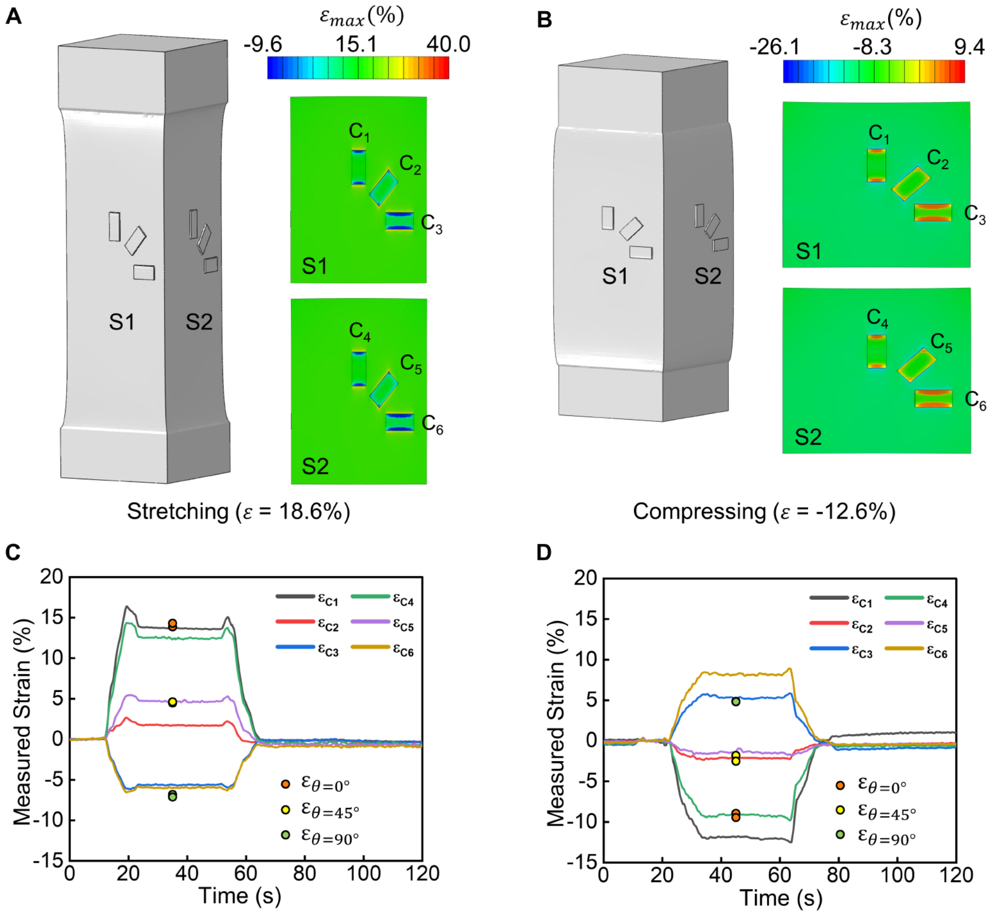

Fig. S24. Comparison of local strain in a deformed soft continuum arm from FEA and experimental measurements using sensors attached to the arm. FEA simulation results show the maximum principal strain distributions of the soft continuum arm under (**A**) 18.6% uniaxial stretching and (**B**) 12.6% uniaxial compressing. The measured local directional strain ε from the distributed sensors based on capacitance responses (data lines: ε_{c1} to ε_{c6}) are compared with the simulated local strain from FEA (solid dots: $\varepsilon_{\theta=0°}$, $\varepsilon_{\theta=45°}$, $\varepsilon_{\theta=90°}$) when the arm is under (**C**) stretching and (**D**) compression.

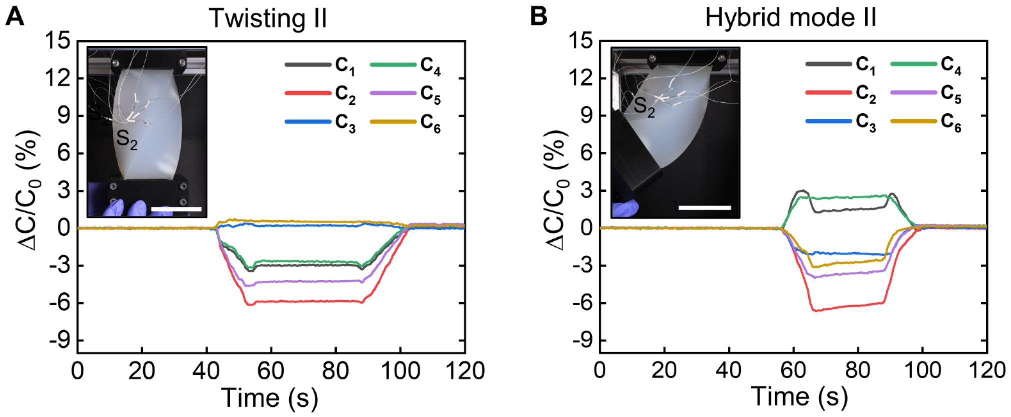

Fig. S25. Additional results of sensing deformations of a soft continuum arm using the distributed sensors under (A) twisting and (B) hybrid deformations. Scale bars, 5 cm.

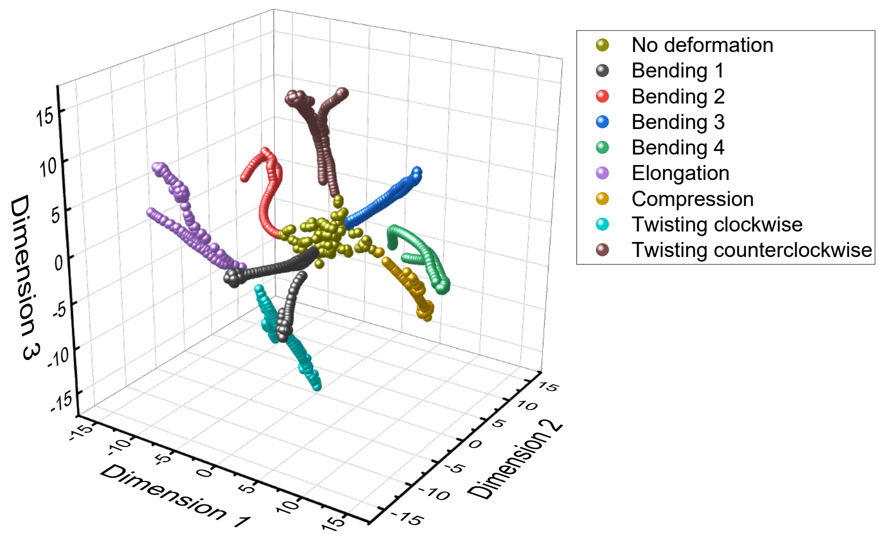

Fig. S26. 3D scatter plot of the capacitance changes collected from 6 sensors in 9 different deformation modes after dimensionality reduction.

**Electrode length**	***L* = 0.5 mm**			***L* = 1.0 mm**		
Prestrain (%)	100	200	300	100	200	300
Measured distance between the bonding sites (μm)	632	460	399	1093	725	593
Measured height (μm)	526	532.6	559.7	904	986	1028
Simulated height (μm)	542	578	586	989.6	1062.8	1086.9

Table S1. Comparison of the heights of origami-inspired 3D electrode structures with two different electrode length designs measured in experiments and FEA.

**Sensor Designs**	**Gauge factor**	**Linearity R^2**	**Hysteresis (%)**	**Baseline capacitance (pF)**	**Number of sensors tested**
Rectangular<br>L = 0.25 mm<br>W = 0.55 mm	-0.205	0.995	5.01	0.68	2
Rectangular<br>L = 0.5mm,<br>W = 1.0mm	-0.25 +/- 0.029	0.997 +/- 0.003	2.42 +/- 0.66	1.51 +/- 0.12	6
Rectangular<br>L = 1.0mm,<br>W = 1.0mm	-0.34 +/- 0.034	0.984 +/- 0.02	3.44 +/- 0.95	2.15 +/- 0.24	5
Trapezoid<br>L = 0.5mm,<br>W = 1.6 mm	-0.274 +/- 0.02	0.996 +/- 0.001	2.65 +/- 1.17	1.91 +/- 0.35	3
Trapezoid<br>L = 1.0 mm,<br>W = 1.8 mm	-0.36	0.978	3.51	2.72	2
5-crease<br>L = 1.0 mm,<br>W = 1.0 mm	-0.44	0.974	4.30	2.41	2

Table S2. Comparison of the gauge factor, linearity, hysteresis, and baseline capacitance values of sensors (under 100% strain) with different electrode designs. The values listed are the average measured values with standard deviation when more than 2 sensors are tested.

Sensing type	Reference	Strain range (%)	Degree of hysteresis (%)	Response time (ms)
**Resistive**	(9)	320	1.02	116
	(37)	100	7.89	120
	(38)	70	1.2	100
	(39)	100	6.2	50
	(40)	100	4.9	344
	(41)	100	2.43	160
**Capacitive**	(42)	100	1	500
	(43)	100	0.35	60
	(44)	100	0.2	90
	(45)	160	3	226
	**This work**	**200**	**1.2**	**22**

Table S3. Comparison of our origami-inspired capacitive strain sensor with other resistive and capacitive stretchable strain sensors on the strain range, degree of hysteresis, and response time.

Supplementary movie captions

Movie S1. Uniaxial stretching and simultaneous capacitance measurement of a representative strain sensor (L = 0.5 mm, rectangular electrodes).

Movie S2. Mechanical robustness tests for the strain sensor.

REFERENCES AND NOTES

1. M. Amjadi, K.-U. Kyung, I. Park, M. Sitti, Stretchable, skin-mountable, and wearable strain sensors and their potential applications: A review. *Adv. Funct. Mater.* **26**, 1678–1698 (2016).

2. S. Wu, K. Moody, A. Kollipara, Y. Zhu, Highly sensitive, stretchable, and robust strain sensor based on crack propagation and opening. *ACS Appl. Mater. Interfaces* **15**, 1798–1807 (2023).

3. H. Wang, M. Totaro, L. Beccai, Toward perceptive soft robots: Progress and challenges. *Adv. Sci.* **5**, 1800541 (2018).

4. M. Wang, T. Wang, Y. Luo, K. He, L. Pan, Z. Li, Z. Cui, Z. Liu, J. Tu, X. Chen, Fusing stretchable sensing technology with machine learning for human-machine interfaces. *Adv. Funct. Mater.* **31**, 2008807 (2021).

5. H. Souri, H. Banerjee, A. Jusufi, N. Radacsi, A. A. Stokes, I. Park, M. Sitti, M. Amjadi, Wearable and stretchable strain sensors: Materials, sensing mechanisms, and applications. *Adv. Intell. Syst.* **2**, 2000039 (2020).

6. N. Matsuhisa, X. Chen, Z. Bao, T. Someya, Materials and structural designs of stretchable conductors. *Chem. Soc. Rev.* **48**, 2946–2966 (2019).

7. D. Y. Choi, M. H. Kim, Y. S. Oh, S.-H. Jung, J. H. Jung, H. J. Sung, H. W. Lee, H. M. Lee, Highly stretchable, hysteresis-free ionic liquid-based strain sensor for precise human motion monitoring. *ACS Appl. Mater. Interfaces* **9**, 1770–1780 (2017).

8. A. Frutiger, J. T. Muth, D. M. Vogt, Y. Mengüç, A. Campo, A. D. Valentine, C. J. Walsh, J. A. Lewis, Capacitive soft strain sensors via multicore-shell fiber printing. *Adv. Mater.* **27**, 2440–2446 (2015).

9. J. Chen, J. Zhang, Z. Luo, J. Zhang, L. Li, Y. Su, X. Gao, Y. Li, W. Tang, C. Cao, Q. Liu, L. Wang, H. Li, Superelastic, sensitive, and low hysteresis flexible strain sensor based on wave-patterned liquid metal for human activity monitoring. *ACS Appl. Mater. Interfaces* **12**, 22200–22211 (2020).

10. S. Yao, Y. Zhu, Wearable multifunctional sensors using printed stretchable conductors made of silver nanowires. *Nanoscale* **6**, 2345–2352 (2014).

11. J. Chen, Q. Yu, X. Cui, M. Dong, J. Zhang, C. Wang, J. Fan, Y. Zhu, Z. Guo, An overview of stretchable strain sensors from conductive polymer nanocomposites. *J. Mater. Chem. C* **7**, 11710–11730 (2019).

12. R. P. Rocha, P. A. Lopes, A. T. de Almeida, M. Tavakoli, C. Majidi, Fabrication and characterization of bending and pressure sensors for a soft prosthetic hand. *J. Micromech. Microeng.* **28**, 034001 (2018).

13. R. L. Truby, C. D. Santina, D. Rus, Distributed proprioception of 3D configuration in soft, sensorized robots via deep learning. *IEEE Robot. Autom. Lett.* **5**, 3299–3306 (2020).

14. M. D. Dickey, Stretchable and soft electronics using liquid metals. *Adv. Mater.* **29**, 1606425 (2017).

15. T. J. Cuthbert, B. C. Hannigan, P. Roberjot, A. V. Shokurov, C. Menon, Hacs: Helical auxetic yarn capacitive strain sensors with sensitivity beyond the theoretical limit. *Adv. Mater.* **35**, 2209321 (2023).

16. S. Yu, H. Yu, Capacitive stretchable strain sensor with low hysteresis based on wavy-shape interdigitated metal electrodes. *IEEE Sens. J.* **21**, 27335–27342 (2021).

17. K. Xu, Y. Lu, S. Honda, T. Arie, S. Akita, K. Takei, Highly stable kirigami-structured stretchable strain sensors for perdurable wearable electronics. *J. Mater. Chem. C* **7**, 9609–9617 (2019).

18. R. Nur, N. Matsuhisa, Z. Jiang, M. O. G. Nayeem, T. Yokota, T. Someya, A highly sensitive capacitive-type strain sensor using wrinkled ultrathin gold films. *Nano Lett.* **18**, 5610–5617 (2018).

19. O. Atalay, A. Atalay, J. Gafford, H. Wang, R. Wood, C. Walsh, A highly stretchable capacitive-based strain sensor based on metal deposition and laser rastering. *Adv. Mater. Technol.* **2**, 1700081 (2017).

20. J. Shintake, T. Nagai, K. Ogishima, Sensitivity improvement of highly stretchable capacitive strain sensors by hierarchical auxetic structures. *Front. Robot. AI* **6**, 127 (2019).

21. K. L. Dorsey, H. Huang, Y. Wen, Origami-patterned capacitor with programmed strain sensitivity. *Multifunct. Mater.* **5**, 025001 (2022).

22. S. Wu, S. Peng, Y. Yu, C.-H. Wang, Strategies for designing stretchable strain sensors and conductors. *Adv. Mater. Technol.* **5**, 1900908 (2020).

23. L. Liu, S. Niu, J. Zhang, Z. Mu, J. Li, B. Li, X. Meng, C. Zhang, Y. Wang, T. Hou, Z. Han, S. Yang, L. Ren, Bioinspired, omnidirectional, and hypersensitive flexible strain sensors. *Adv. Mater.* **34**, e2200823 (2022).

24. J. Rogers, Y. Huang, O. G. Schmidt, D. H. Gracias, Origami MEMS and NEMS. *MRS Bull.* **41**, 123–129 (2016).

25. Y. Feng, Z. Zhou, W. Wang, Z. Rao, Y. Han, The 3D capacitance modeling of non-parallel plates based on conformal mapping, *IEEE NEMS*, 1264–1267.

26. Y. Xiang, Further study on electrostatic capacitance of an inclined plate capacitor. *J. Electrostat.* **66**, 366–368 (2008).

27. S. Xu, Z. Yan, K.-I. Jang, W. Huang, H. Fu, J. Kim, Z. Wei, M. Flavin, J. McCracken, R. Wang, A. Badea, Y. Liu, D. Xiao, G. Zhou, J. Lee, H. U. Chung, H. Cheng, W. Ren, A. Banks, X. Li, U. Paik, R. G. Nuzzo, Y. Huang, Y. Zhang, J. A. Rogers, Assembly of micro/nanomaterials into complex, three-dimensional architectures by compressive buckling. *Science* **347**, 154–159 (2015).

28. Z. Yan, F. Zhang, J. Wang, F. Liu, X. Guo, K. Nan, Q. Lin, M. Gao, D. Xiao, Y. Shi, Y. Qiu, H. Luan, J. H. Kim, Y. Wang, H. Luo, M. Han, Y. Huang, Y. Zhang, J. A. Rogers, Controlled mechanical buckling for origami-inspired construction of 3D microstructures in advanced materials. *Adv. Funct. Mater.* **26**, 2629–2639 (2016).

29. H. Zhao, K. Li, M. Han, F. Zhu, A. Vázquez-Guardado, P. Guo, Z. Xie, Y. Park, L. Chen, X. Wang, H. Luan, Y. Yang, H. Wang, C. Liang, Y. Xue, R. D. Schaller, D. Chanda, Y. Huang, Y. Zhang, J. A. Rogers, Buckling and twisting of advanced materials into morphable 3D mesostructures. *Proc. Natl. Acad. Sci. U.S.A.* **116**, 13239–13248 (2019).

30. X. Ning, X. Wang, Y. Zhang, X. Yu, D. Choi, N. Zheng, D. S. Kim, Y. Huang, Y. Zhang, J. A. Rogers, Assembly of advanced materials into 3D functional structures by methods inspired by origami and kirigami: A review. *Adv. Mater. Interfaces* **5**, 1800284 (2018).

31. *Physical Properties of Glycerine and Its Solutions*, Glycerine Producers' Association (1963).

32. Y. Hotta, Y. Zhang, N. Miki, A flexible capacitive sensor with encapsulated liquids as dielectrics. *Micromachines* **3**, 137–149 (2012).

33. W. N. Sharpe Jr., J. Pulskamp, B. G. Mendis, C. Eberl, D. S. Gianola, R. Polcawich, K. J. Hemker, Tensile stress-strain curves of gold film, *ASME 2006 International Mechanical Engineering Congress and Exposition* (ASME, 2006), pp. 533–540.

34. C.-W. Baek, J.-M. Kim, Y.-K. Kim, J. H. Kim, H. J. Lee, S. W. Han, Mechanical characterization of gold thin films based on strip bending and nanoindentation test for mems/nems applications. *Sens. Mater.* **17**, 277–288 (2005).

35. I. Chasiotis, C. Bateson, K. Timpano, A. S. McCarty, N. S. Barker, J. R. Stanec, Strain rate effects on the mechanical behavior of nanocrystalline Au films. *Thin Solid Films* **515**, 3183–3189 (2007).

36. R. S. Lakes, *Viscoelastic Materials* (Cambridge Univ. Press, 2009).

37. A. S. Kurian, H. Souri, V. B. Mohan, D. Bhattacharyya, Highly stretchable strain sensors based on polypyrrole-silicone rubber composites for human motion detection. *Sens. Actuator A Phys.* **312**, 112131 (2020).

38. Q. Xia, S. Wang, W. Zhai, C. Shao, L. Xu, D. Yan, N. Yang, K. Dai, C. Liu, C. Shen, Highly linear and low hysteresis porous strain sensor for wearable electronic skins. *Compos. Commun.* **26**, 100809 (2021).

39. A. Yoshida, Y.-F. Wang, T. Sekine, Y. Takeda, D. Kumaki, S. Tokito, Printed low-hysteresis stretchable strain sensor based on a self-segregating conductive composite. *ACS Appl. Eng. Mater.* **1**, 50–58 (2023).

40. J.-H. Pu, X. Zhao, X.-J. Zha, L. Bai, K. Ke, R.-Y. Bao, Z.-Y. Liu, M.-B. Yang, W. Yang, Multilayer structured agnw/wpu-mxene fiber strain sensors with ultrahigh sensitivity and a wide operating range for wearable monitoring and healthcare. *J. Mater. Chem. A* **7**, 15913–15923 (2019).

41. S. Ko, A. Chhetry, D. Kim, H. Yoon, J. Y. Park, Hysteresis-free double-network hydrogel-based strain sensor for wearable smart bioelectronics. *ACS Appl. Mater. Interfaces* **14**, 31363–31372 (2022).

42. D. Zhang, J. Zhang, Y. Wu, X. Xiong, J. Yang, M. D. Dickey, Liquid metal interdigitated capacitive strain sensor with normal stress insensitivity. *Adv. Intell. Syst.* **4**, 2100201 (2022).

43. X. Wang, Y. Deng, P. Jiang, X. Chen, H. Yu, Low-hysteresis, pressure-insensitive, and transparent capacitive strain sensor for human activity monitoring. *Microsyst. Nanoeng.* **8**, 113 (2022).

44. J. S. Meena, S. B. Choi, T. D. Khanh, H. S. Shin, J. S. Choi, J. Joo, J.-W. Kim, Highly stretchable and robust textile-based capacitive mechanical sensor for human motion detection. *Appl. Surf. Sci.* **613**, 155961 (2023).

45. N. J. Kumar, A. Johnson, D. Roggen, N. Münzenrieder, A flexible capacitance strain sensor with stitched contact terminals. *Adv. Mater. Technol.* **8**, 2200410 (2023).

46. Z. Shen, Z. Zhang, N. Zhang, J. Li, P. Zhou, F. Hu, Y. Rong, B. Lu, G. Gu, High-stretchability, ultralow-hysteresis conductingpolymer hydrogel strain sensors for soft machines. *Adv. Mater.* **34**, 2203650 (2022).

47. Y. Zhang, X. Zhu, Y. Liu, L. Liu, Q. Xu, H. Liu, W. Wang, L. Chen, Ultra-stretchable monofilament flexible sensor with low hysteresis and linearity based on MWCNTs/Ecoflex composite materials. *Macromol. Mater. Eng.* **306**, 2100113 (2021).

48. X. Hu, F. Yang, M. Wu, Y. Sui, D. Guo, M. Li, Z. Kang, J. Sun, J. Liu, A super-stretchable and highly sensitive carbon nanotube capacitive strain sensor for wearable applications and soft robotics. *Adv. Mater. Technol.* **7**, 2100769 (2022).

49. G. Cai, J. Wang, K. Qian, J. Chen, S. Li, P. S. Lee, Extremely stretchable strain sensors based on conductive self-healing dynamic cross-links hydrogels for human-motion detection. *Adv. Sci.* **4**, 1600190 (2017).

50. Z. Liao, M. Hossain, X. Yao, R. Navaratne, G. Chagnon, A comprehensive thermo-viscoelastic experimental investigation of Ecoflex polymer. *Polym. Test.* **86**, 106478 (2020).

51. R. K. Sahu, K. Patra, Rate-dependent mechanical behavior of VHB 4910 elastomer. *Mech. Adv. Mater. Struct.* **23**, 170–179 (2016).

52. B. Aksoy, Y. Hao, G. Grasso, K. M. Digumarti, V. Cacucciolo, H. Shea, Shielded soft force sensors. *Nat. Commun.* **13**, 4649 (2022).

53. J. Tapia, E. Knoop, M. Mutný, M. A. Otaduy, M. Bächer, Makesense: Automated sensor design for proprioceptive soft robots. *Soft Robot.* **7**, 332–345 (2020).

54. D. Hu, F. Giorgio-Serchi, S. Zhang, Y. Yang, Stretchable e-skin and transformer enable high-resolution morphological reconstruction for soft robots. *Nat. Mach. Intell.* **5**, 261–272 (2023).

55. I. M. Van Meerbeek, C. M. De Sa, R. F. Shepherd, Soft optoelectronic sensory foams with proprioception. *Sci. Robot.* **3**, eaau2489 (2018).

56. J. H. Lee, S. H. Kim, J. S. Heo, J. Y. Kwak, C. W. Park, I. Kim, M. Lee, H.-H. Park, Y.-H. Kim, S. J. Lee, S. K. Park, Heterogeneous structure omnidirectional strain sensor arrays with cognitively learned neural networks. *Adv. Mater.* **35**, e2208184 (2023).

57. S. H. Kim, H. S. Chang, C. H. Shih, N. K. Uppalapati, U. Halder, G. Krishnan, P. G. Mehta, M. Gazzola, A physics-informed, vision-based method to reconstruct all deformation modes in slender bodies. *2022 IEEE International Conference on Robotics and Automation (ICRA)*, 4810–4817 (2022).

58. C. A. Neugebauer, Tensile properties of thin, evaporated gold films. *J. Appl. Phys.* **31**, 1096–1101 (1960).

59. F. E. H. Tay, J. Xu, Y. C. Liang, V. J. Logeeswaran, Y. Yao, The effects of non-parallel plates in a differential capacitive microaccelerometer. *J. Micromech. Microeng.* **9**, 283 (1999).

60. D. Kim, D. Y. Kim, J. Shim, K. C. Kim, Energy harvesting performance of an edlc power generator based on pure water and glycerol mixture: Analytical modeling and experimental validation. *Sci. Rep.* **11**, 23426 (2021).
